# Orf Virus ORF120 Protein Positively Regulates the NF-κB Pathway by Interacting with G3BP1

**DOI:** 10.1128/JVI.00153-21

**Published:** 2021-09-09

**Authors:** Yanlong Zhou, Jiyu Guan, Feng Gao, Zi Li, Yungang Lan, Huijun Lu, Deguang Song, Lijun Lv, Pin Lv, Mengshi Xu, Zhenzhen Wang, Hongbin He, Kui Zhao, Wenqi He

**Affiliations:** a Key Laboratory of Zoonosis, Ministry of Education, College of Veterinary Medicine, Jilin University, Changchun, China; b Key Laboratory of Zoonosis, Ministry of Education, Institute of Zoonosis, Jilin University, Changchun, China; c Key Laboratory of Animal Resistant Biology of Shandong, Ruminant Disease Research Center, College of Life Sciences, Shandong Normal University, Jinan, China; University of Illinois at Urbana Champaign

**Keywords:** ORFV, ORF120 protein, G3BP1, NF-κB signaling, antiviral immune response, evasion mechanism

## Abstract

Orf virus (ORFV) is a highly epitheliotropic parapoxvirus with zoonotic significance that induces proliferative lesions in the skin of sheep, goats, and humans. Several viral proteins carried by ORFV, including nuclear factor-κB (NF-κB) inhibitors, play important roles in hijacking host-associated proteins for viral evasion of the host innate immune response. However, the roles of proteins with unknown functions in viral replication and latent infection remain to be explored. Here, we present data demonstrating that the ORF120, an early-late ORFV-encoded protein, activates the NF-κB pathway in the early phase of infection, which implies that ORFV may regulate NF-κB through a biphasic mechanism. A DUAL membrane yeast two-hybrid system and coimmunoprecipitation experiments revealed that the ORF120 protein interacts with Ras-GTPase-activating protein (SH3 domain) binding protein 1 (G3BP1). The overexpression of the ORF120 protein can efficiently increase the expression of G3BP1 and nuclear translocation of NF-κB-p65 in primary ovine fetal turbinate (OFTu) and HeLa cells. The knockdown of G3BP1 significantly decreased ORF120-induced NF-κB activation, indicating that G3BP1 is involved in ORF120-induced NF-κB pathway activation. A dual-luciferase reporter assay revealed that ORF120 could positively regulate the NF-κB pathway through the full-length G3BP1 or the domain of G3BP1^RRM+RGG^. In conclusion, we demonstrate, for the first time, that the ORF120 protein is capable of positively regulating NF-κB signaling by interacting with G3BP1, providing new insights into ORFV pathogenesis and a theoretical basis for antiviral drug design.

**IMPORTANCE** As part of the host innate response, the nuclear factor-κB (NF-κB) pathway plays a partial antiviral role in nature by regulating the innate immune response. Thus, the NF-κB pathway is probably the most frequently targeted intracellular pathway for subversion by anti-immune modulators that are carried by a wide range of pathogens. Various viruses, including poxviruses, carry several proteins that prepare the host cell for viral replication by inhibiting cytoplasmic events, leading to the initiation of NF-κB transcriptional activity. However, NF-κB activity is hypothesized to facilitate viral replication to a great extent. The significance of our research is in the exploration of the activation mechanism of NF-κB induced by the Orf virus (ORFV) ORF120 protein interacting with G3BP1, which helps not only to explain the ability of ORFV to modulate the immune response through the positive regulation of NF-κB but also to show the mechanism by which the virus evades the host innate immune response.

## INTRODUCTION

Orf virus (ORFV) belongs to the *Parapoxvirus* genus of the *Poxviridae* family and often infects sheep, goats, and other ruminants around the world (1). ORFV is an epitheliotropic linear double-stranded DNA virus that causes highly contagious vesiculoulcerative pustular and self-limiting skin lesions in sheep and goats, known as contagious ecthyma (2). It can be transmitted to humans, particularly shepherds, farmers, butchers, and veterinarians, in direct or indirect contact with infected animals (3).

An analysis of the complete genomic sequences of ORFV has revealed several genes located at terminal regions capable of modulating the host response (4). Among these genes, many encode viral immune regulators identified as soluble versions of cellular cytokine receptors. ORFV OV20.0 protein, an ortholog of the vaccinia virus (VACV) E3, with a similar innate immune evasion mechanism, can interact with PKR and its two known activators, double-stranded DNA (dsRNA) and the cellular PKR activator (PACT), thus establishing efficient viral infection by inhibiting PKR activation (5). A recent study confirmed that the OV20.0 protein can directly bind to the dsRNA binding domains of adenosine deaminase acting on RNA 1 (ADAR1). The OV20.0 protein might evade antiviral responses via PKR by modulating ADAR1-dependent gene expression (6). A viral ortholog of mammalian interleukin-10 (vIL-10) is an anti-inflammatory cytokine (7). The chemokine-binding protein (CBP) encoded by the ORF 112 gene can block immune cell recruitment to the sites of infection by disrupting chemokine gradients (8). A novel inhibitor of the cytokines granulocyte-macrophage colony-stimulating factor (GM-CSF) and interleukin-2 (IL-2; GIF), an intermediate-late viral protein encoded in several strains of ORFV, binds to and inhibits the ovine cytokines GM-CSF and IL-2, thus disrupting host immune and inflammatory responses (9). Vascular endothelial growth factor-E (VEGF-E), found in the genome of ORFV, specifically binds to VEGF receptor-2 (VEGFR-2) and mediates mitotic activity in endothelial cells (10). A viral Bcl-2-like protein (ORFV 125) has been confirmed to function in a Bcl-2 manner to inhibit apoptosis (11). Recently, a growing number of ORFV immunomodulators (ORFV002, ORFV024, ORFV073, ORFV119, and ORFV121) were found to be involved in the inhibition of the nuclear factor-κB (NF-κB) pathway, thus modulating the immune response (12).

NF‐κB is an inducible transcription factor typically activated by proinflammatory cytokines and other specific stimuli and is mainly involved in the regulation of inflammatory and immune processes, including innate and adaptive immunity (13, 14). The NF‐κB pathway has been demonstrated to be important in antiviral responses; however, many viruses have evolved sophisticated mechanisms to regulate NF‐κB signaling pathways by deploying subversive proteins or hijacking host signaling molecules, thus allowing viruses to evade and subvert the host immune response (15, 16). Larger DNA viruses that have a complex genome, such as herpesviruses, adenoviruses, and poxviruses, can carry multiple viral proteins that inhibit NF‐κB activation. For example, African swine fever virus (ASFV) carries a viral homolog of IκBα (NF‐κB inhibitor α), A238L, which interacts with cellular RELA and thus suppresses the activation of NF-κB complexes (17, 18). The immediate-early protein of herpes simplex virus (HSV), ICP0, reduces Toll-like receptor 2 (TLR2)-mediated inflammatory responses against the virus by inducing degradation of the MYD88 adaptor (19). Another immediate-early protein of HSV, ICP27, inhibits NF-κB activity by stabilizing IκBα through blockade of its phosphorylation and ubiquitylation at the early stage of infection (20). The adenovirus E3 10.4 kDa/14.5 kDa protein complex can inhibit tumor necrosis factor alpha (TNF-α)-mediated activation of the IκB kinase (IKK) complex (21). Vaccinia virus (VACV) carries six known NF-κB regulators, namely, A46R, A52R, B14, K1L, M2L, and N1L, of which all block the activation of the IKK complex and inhibit the degradation of IκBα via diverse mechanisms (22–28). ORFV has evolved novel mechanisms to counteract the NF-κB signaling pathway at different steps by targeting cytoplasmic activation events involving NF-κB pathways (29–33).

G3BP1, also known as stress granule assembly factor 1, is a ubiquitously expressed cytosolic protein that was originally identified as a binding partner of Ras GTPase-activating protein (GAP). As a multidomain protein, G3BP1 contains a nuclear transporter factor 2 (NTF2) domain, acidic domain, PxxP domain, RNA-recognition module (RRM), and arginine-glycine-glycine (RGG) motif, which has been identified to be evolutionarily conserved from yeast to humans (34, 35). Responding to several types of cellular stresses, such as DNA damage, oxidative stress, and viral infection, G3BP1 is a core component of stress granules (SGs), which are critical for nucleating SG assembly (36). G3BP1 has also been reported to have an antiviral role during many viral infections, often activating the innate immune response through NF‐κB and Jun N-terminal protein kinase (JNK) transcription (35, 37–39). A recent study demonstrates that G3BP1 is pivotal for the efficient activation of cyclic GMP-AMP (cGAS) and for DNA sensing (40). Apart from its critical role in regulating cGAS-mediated immune responses, G3BP1 can also bind to viral dsRNA and RIG-I to enhance the interferon beta (IFN-β) response, thus reducing virus replication (41).

Although viruses more often inhibit NF-κB signaling pathway activation to escape the host immune response, some viruses regulate NF-κB in a biphasic manner to optimize viral replication in infected cells (15). In the present study, we demonstrated by immunostaining that the ORF120 protein, an ORFV-encoded early-late protein, localizes in both the cytoplasm and nucleus and is able to activate the NF-κB pathway in the early phase of infection by phosphorylating the IκB kinase (IKK) complex, degrading IκBα and phosphorylating and inducing the nuclear translocation of NF-κB-p65. In addition, the ORF 120 protein was confirmed to regulate the NF-κB pathway by interacting with G3BP1 during ORFV infection. The NTF2 and PxxP domains of G3BP1 were identified as the potential ORF120-G3BP1 binding sites by the immunoprecipitation of HEK293T cells overexpressing GFP-ORF120 or hemagglutinin (HA)-ORF120 together with truncated G3BP1. A dual-luciferase reporter assay revealed that ORF120 could positively regulate the NF-κB pathway through the full-length G3BP1 or the domain of G3BP1^RRM+RGG^. Taken together, our results provide new insight into host-pathogen interactions that regulate the NF-κB pathway during ORFV infection.

## RESULTS

### The generation of the OV-SY17Δ120 deletion mutant and RFP-tagged OV-SY17-RV120 revertant viruses using reporter genes as selection markers and the analysis of their growth kinetics.

**FIG 1:**
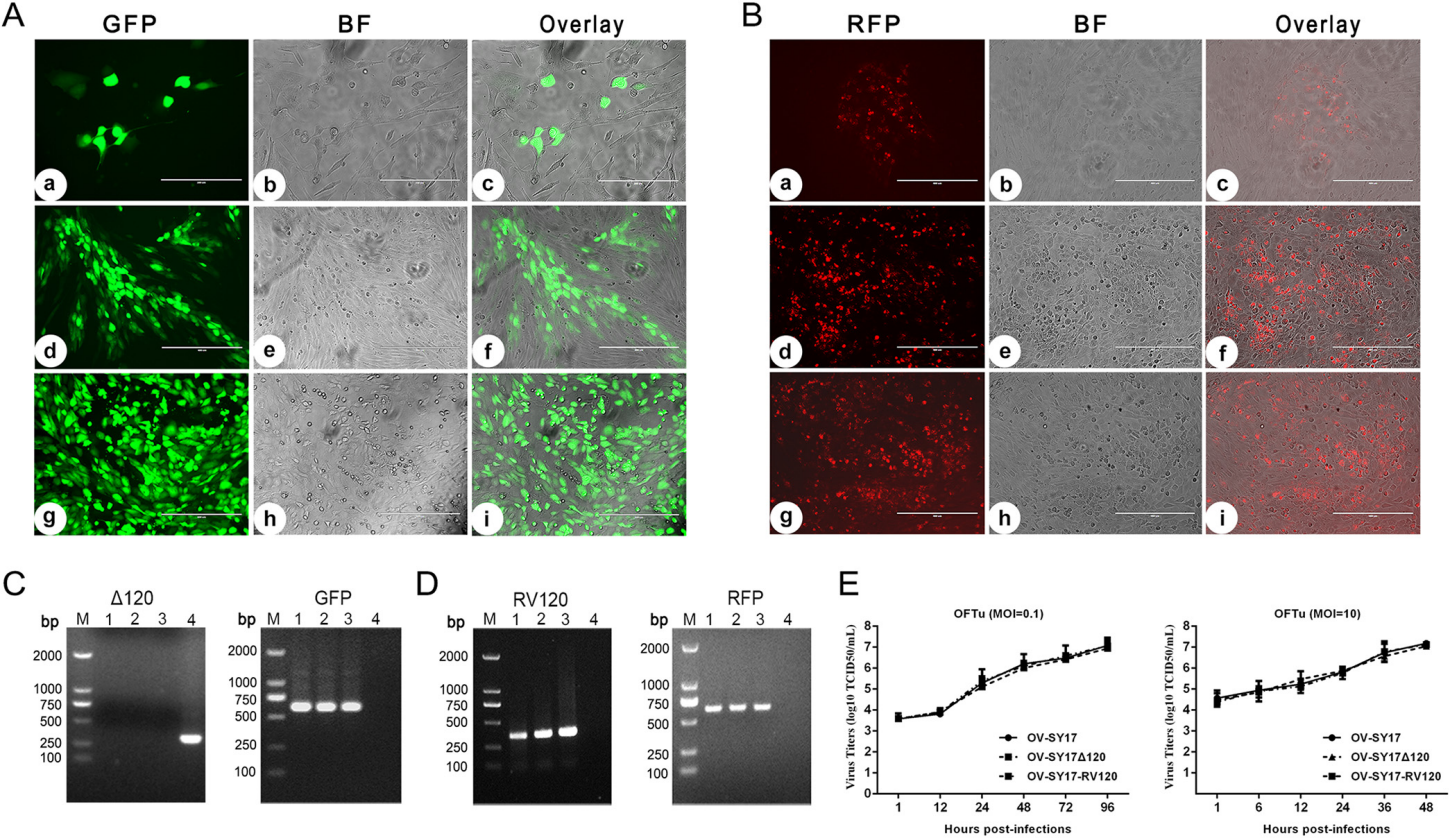
Generation of the recombinant viruses and their growth properties *in vitro*. (A) Fluorescence microscopy showing the cytopathic effects (CPEs) of the OFTu cells infected with OV-SY17Δ120 deletion mutant virus by plaque purification. The plaques with a strong fluorescent signal in the OFTu cells infected with OV-SY17Δ120 48 hpi during the second round of plaque purification. (b, e, and h) The same fields as shown in a, d, and g by bright-field microscopy. (c, f, and i) Overlay of the a and b, d and e, or g and h images. The cells were visualized under a microscope at 10× magnification. Bar, 400 μm. (B) A revertant mutant OV-SY17-RV120 was produced using red fluorescent protein (RFP) as a selection marker and obtained after two rounds of plaque assay purification on monolayers of OFTu cell cultures 48 hpi. (b, e, and h) The same fields as in a, d, and g by bright-field microscopy. (c, f, and i) Overlay of the a and b, d and e, or g and h images. The cells were visualized under a microscope at 10× magnification. Bar, 400 μm. (C) PCR was performed to confirm the absence of ORFV ORF120 and the presence of the EGFP reporter gene sequences in the OV-SY17Δ120 genome. The PCR analysis showed that the ORF120 gene was successfully deleted in the OV-SY17Δ120 mutant. M, molecular marker; lanes 1 to 3, three OV-SY17Δ120 recombinants from plaque purification; lane 4, wild-type OV-SY17. (D) PCR was performed to confirm the presence of both the ORF120 and RFP reporter genes in the OV-SY17-RV120 genome. The PCR analysis showed that the corresponding revertant virus OV-SY17-RV120 was successfully rescued. M, molecular marker; lanes 1 to 3, three OV-SY17-RV120 recombinants from plaque purification; lane 4, wild-type OV-SY17. (E) *In vitro* growth kinetics of the OV-SY17Δ120 mutant virus, OV-SY17 parental virus, and OV-SY17-RV120 revertant virus. Primary OFTu cells were infected (MOI, 10 or 0.1) with either the OV-SY17Δ120 mutant virus, parental virus OV-SY17, or revertant virus OV-SY17-RV120 and the virus titers were determined by TCID_50_ assays at the indicated postinfection time points. The growth curves of the OV-SY17Δ120 mutant virus were similar to those of the OV-SY17 parental virus and the OV-SY17-RV120 revertant virus, indicating that the deletion of ORF120 did not affect virus replication.

To better understand the role of the ORF120 protein in ORFV-infected cells, we generated recombinant viruses lacking the coding sequence (CDS) of the ORF120 gene. The ORF120 gene was deleted from the OV-SY17 virus and replaced with a cassette containing an enhanced green fluorescent protein (EGFP) reporter under the control of the early-late VV7.5 promoter by homologous recombination. The recombinant virus that formed GFP-positive plaques was selected and obtained by successive rounds of plaque assay purification on monolayers of primary ovine fetal turbinate (OFTu) cell cultures (Fig. 1A). The virus population obtained from the last round of plaque purification was amplified in primary OFTu cell cultures to obtain a virus stock. The purity of OV-SY17Δ120 in the virus stock was assessed by PCR using primers targeting ORF120 and EGFP genes to ensure the absence of parental OV-SY17. Only amplicons for the EGFP gene were detected in the DNA extracted from the virus stock; no amplicons were generated with primers targeting the ORF120 gene (Fig. 1C). Also, ORF120 was confirmed to be replaced by EGFP in the OV-SY17Δ120 deletion mutant using whole-genome sequencing (data not shown), indicating that the OV-SY17Δ120 stock was not contaminated with OV-SY17 and that GFP was inserted only in the right location. Furthermore, a revertant mutant, OV-SY17-RV120, was produced using red fluorescent protein (RFP) as a selection marker and obtained by successive rounds of plaque assay purification on monolayers of OFTu cell cultures (Fig. 1B). The purity of OV-SY17-RV120 in the virus stock was assessed by PCR using primers targeting the ORF120 and RFP genes. Positive amplification results were observed in DNA extracted from the revertant virus stock using primers targeting ORF120 and RFP (Fig. 1D). The *in vitro* growth characteristics of the OV-SY17Δ120 mutant virus obtained were evaluated in primary OFTu cells based on multistep and one-step growth curves and compared with those of the parental virus OV-SY17 or the RFP-tagged revertant virus OV-SY17-RV120. The replication kinetics of OV-SY17Δ120 were not significantly different from those of the parental virus OV-SY17 or the revertant virus OV-SY17-RV120 (Fig. 1E), indicating that the deletion of the ORF120 gene in ORFV did not affect the ability of the virus to replicate in OFTu cells. Thus, we confirmed that the ORF120 gene was not required for the replication of this virus in cell culture.

### ORFV ORF120 is transcribed both early and late during the infection and localizes to the cell cytoplasm and nucleus.

**FIG 2:**
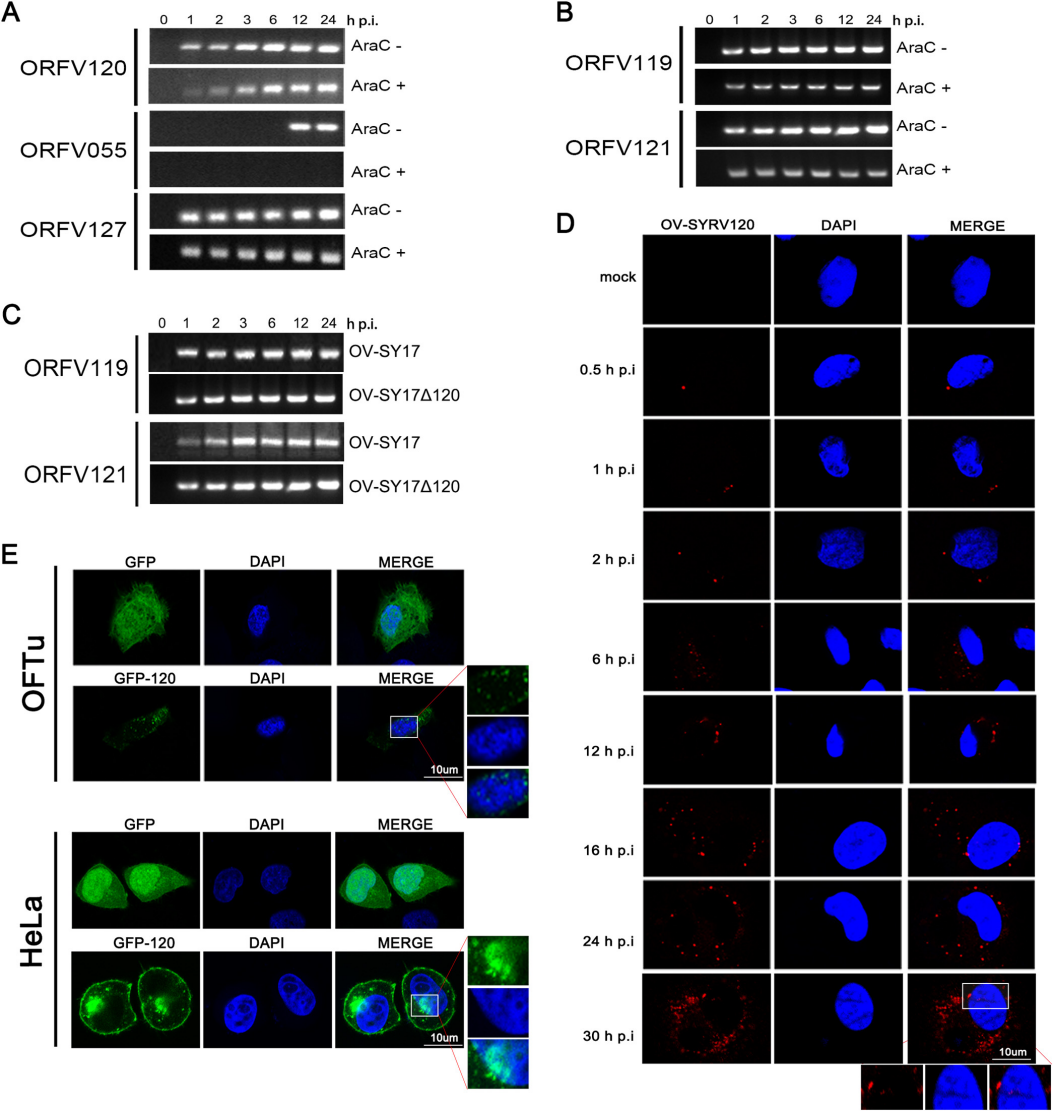
Transcription kinetics of the ORF120 gene and the intracellular localization of the ORF120 protein. (A) The transcription kinetics of ORF120 during the viral life cycle in the presence or absence of AraC were detected through RT-PCR. The transcript levels of ORF120 were detected by RT-PCR as early as 1 hpi, and ORF120 transcription levels continued to be detected with time throughout the 24 h period in the presence or absence of AraC. (B) The deletion of ORF120 in the GFP-labeled OV-SY17Δ120 mutant did not affect the transcription of neighboring genes, including ORFV119 and ORFV121. (C) The transcription of two neighboring genes of ORF120 in parental virus OV-SY17 and OV-SY17-Δ120 deletion mutant was not affected by the absence of ORF120. (D) The intracellular localization of the ORF120 protein in the OFTu cells infected with RFP-labeled OV-SY17-RV120 revertant mutant and in the OFTu cells and HeLa cells overexpressing the GFP-ORF120 fusion protein. Bright red fluorescence was observed in the cytoplasm of the OV-SY17-RV120-infected cells, showing a spotty pattern and increasing fluorescence intensity. Meanwhile, a very small amount of ORF120 protein was observed in the nucleus. (E) Bright-green fluorescence was observed mainly in the cytoplasm of the ORF120-overexpressing OFTu cells and HeLa cells and distributed along the nuclear membrane and cell membrane, forming a ring-like structure. Additionally, a very small amount of ORF120 protein was detected in the nucleus.

To determine the transcription kinetics of the ORFV ORF120 gene during the viral life cycle, cells were infected with virus in the presence or absence of AraC. As shown in Fig. 2A, the transcription levels of ORF120 were detected by reverse transcription-PCR (RT-PCR) as early as 1 hour postinfection (hpi), and ORF120 transcription levels continued to be detected throughout the 24-h period in the presence or absence of AraC. Similarly, the transcription of ORFV127 (an early viral gene) showed similar kinetics. In contrast, the transcription of ORFV055 (a late viral gene) was blocked by the presence of AraC (Fig. 2A). Because AraC can slightly inhibit ORF120 mRNA synthesis and ORF120 mRNA is detected throughout the course of infection, ORF120 is a gene that expresses during both early and late times during the infection. Additionally, the transcription levels of neighboring genes, including ORFV119 and ORFV121, have been demonstrated by RT-PCR as not affected in the ORF120-deletion mutant (Fig. 2B). Also, the transcription of two neighboring genes of ORF120 in parental virus OV-SY17 and the OV-SY17-Δ120 deletion mutant was not affected by the absence of ORF120 (Fig. 2C). To determine the intracellular localization of the ORFV ORF120 protein in infected cells, OFTu cells were mock infected or infected with the red fluorescent protein (RFP)-labeled OV-SY17-RV120 revertant mutant. Compared with uninfected cells, bright red fluorescence was observed in the cytoplasm of the infected cells, showing a spotty pattern. For 24 h, OV-SY17-RV120-infected cells showed an increase in fluorescence intensity (Fig. 2D). Meanwhile, a very small amount of the ORF120 protein was observed in the nucleus. Further studies on the subcellular localization of the ORF120 protein were performed in OFTu cells and HeLa cells overexpressing the GFP-ORF120 fusion protein. Bright green fluorescence in a ring-like pattern was observed mainly in the cytoplasm of the ORF120-overexpressing OFTu cells. Additionally, a very small amount of the ORF120 protein was detected in the nucleus (Fig. 2E). A similar distribution pattern was observed for GFP-ORF120 in the ORF120-overexpressing HeLa cells (Fig. 2E).

### ORFV ORF120 protein enhances the transcription of NF-κB-mediated genes during infection.

**FIG 3:**
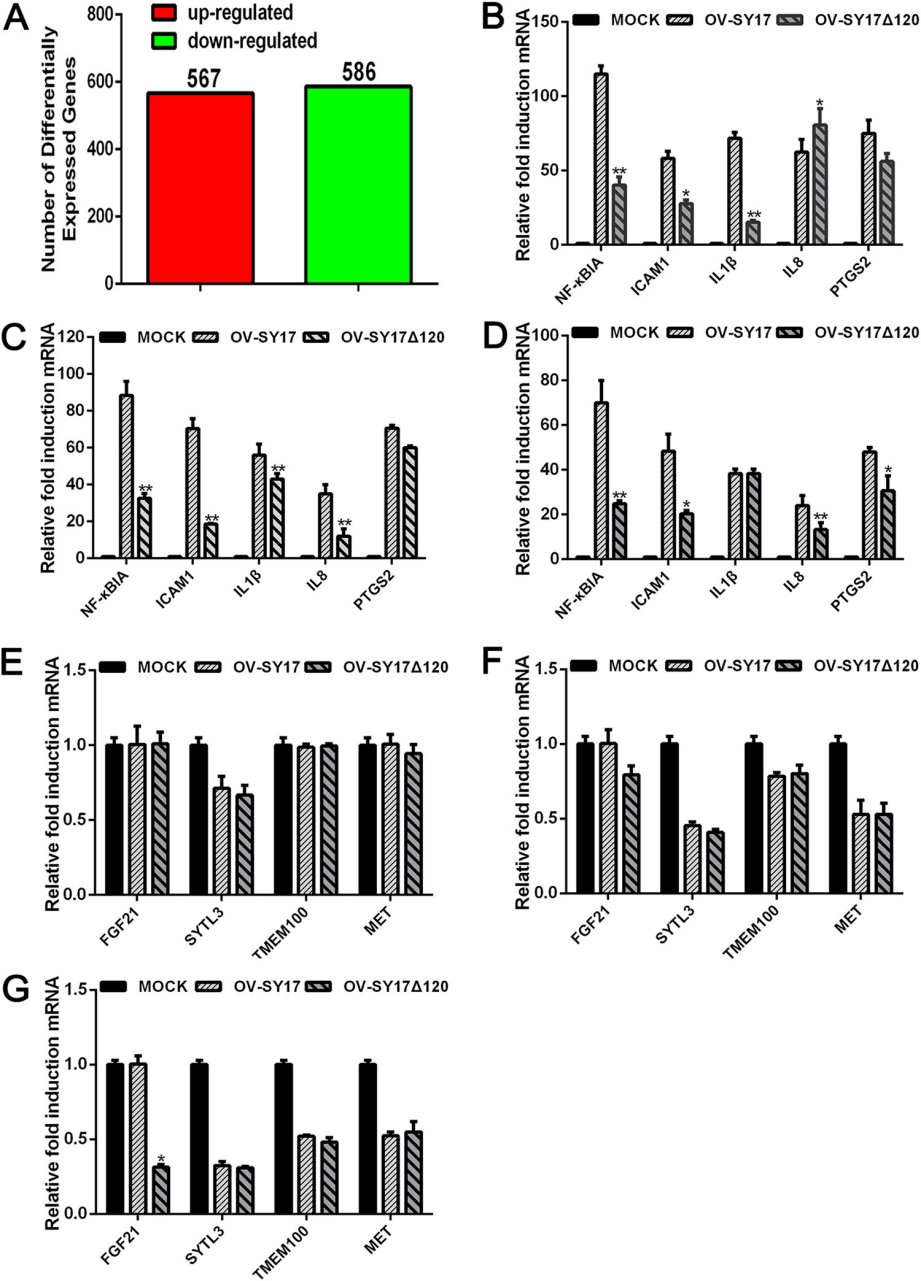
Induced effect of the ORF120 protein on the transcription of the NF-κB-regulated and non-NF-κB-regulated genes. (A) Differentially expressed genes analyzed by digital gene expression profiling and transcriptome analysis. (B to D) The mRNA expression levels of NF-κBIA, ICAM1, IL-1β, IL-8, and PTGS2 mRNA in OFTu cells infected with OV-SY17 and OV-SY17Δ120 mutant (MOI, 10) were quantified by qPCR at 1, 2, and 3 hpi. The fold changes are shown relative to the levels after the OV-SY17 treatment. The results are the mean values of three independent experiments (*, *P < *0.05; **, *P* < 0.01). (E to G) The mRNA expression levels of FGF21, SYTL3, TMEM100, and MET mRNA in OFTu cells infected with OV-SY17 and OV-SY17Δ120 mutant (MOI, 10) were quantified by qPCR at 1, 2, and 3 hpi. The fold changes are shown relative to the levels after the OV-SY17 treatment. The results are the mean values of three independent experiments (*, *P < *0.05; **, *P* < 0.01).

Transcriptome sequencing (RNA-seq) of cells infected with the OV-SY17 wild-type virus or the OV-SY17Δ120 ORF120-deletion mutant virus was performed 3 hpi. A total of 1,153 differentially expressed genes (DEGs) were obtained, including 567 upregulated and 586 downregulated genes (Fig. 3A). The data set containing the raw reads was deposited in the NCBI Sequence Read Archive (SRA) database (accession number PRJNA675661). The functional similarities of these DEGs were assessed through enrichment analysis based on Gene Ontology (GO) terms. The DEGs were mainly enriched for several biological functions, including nucleic acid binding, transcription factor activity, translation regulator activity, signal transducer activity, morphogen activity, response to stimulus, immune system process, and metabolic process. Furthermore, an initial screening revealed that the expression levels of NF-κB-mediated proinflammatory cytokines decreased in the OV-SY17Δ120-infected cells (Table 1).

**TABLE 1:** Summary of NF-κB-regulated genes detected by transcriptome sequencing in primary OFTu cells infected with OV-SY17 vs OV-SY-Δ120

Gene	Description	GenBank	log2FC^*a*^
NF-κBIA	Nuclear factor of kappa light polypeptidase gene enhancer in B-cell inhibitor, alpha	NM_001045868	−1.67577
ICAM1	Intercellular adhesion molecule (CD54)	NM_174348	−2.35203
IL-8	IL-8	NM_173925	−3.143662
CCL20	Chemokine ligand 20	NM_174263	−5.09038
PTGS2	Prostaglandin-endoperoxidase synthase 2	NM_17445	−3.739541

a FC, fold change.

Based on the comparative transcriptome analysis results, five differentially expressed transcripts mediated by NF-κB, including the proinflammatory cytokines (IL-1β and IL-8) and the proinflammatory mediators (NF-κBIA, ICAM1, and PTGS2), were selected and further analyzed by quantitative real-time PCR (qPCR) at 1, 2, and 3 hpi (Fig. 3B to D). The cells expressing the OV-SY17Δ120 mutant showed significantly decreased expression levels of NF-κB-mediated genes compared with the cells expressing the wild-type OV-SY17 virus. However, the mRNA expression levels of some non-NF-κB-mediated genes, such as SYTL3, TMEM100, and MET, in the cells expressing the OV-SY17Δ120 mutant and the cells expressing the wild-type OV-SY17 virus showed no significant change; only FGF21 showed significantly decreased expression levels at 3 hpi (Fig. 3E to G). These results indicated that the ORF120 protein can induce the transcription of NF-κB-mediated genes during infection.

### Transcriptional activation of NF-κB is induced by the ORFV ORF120 protein.

**FIG 4:**
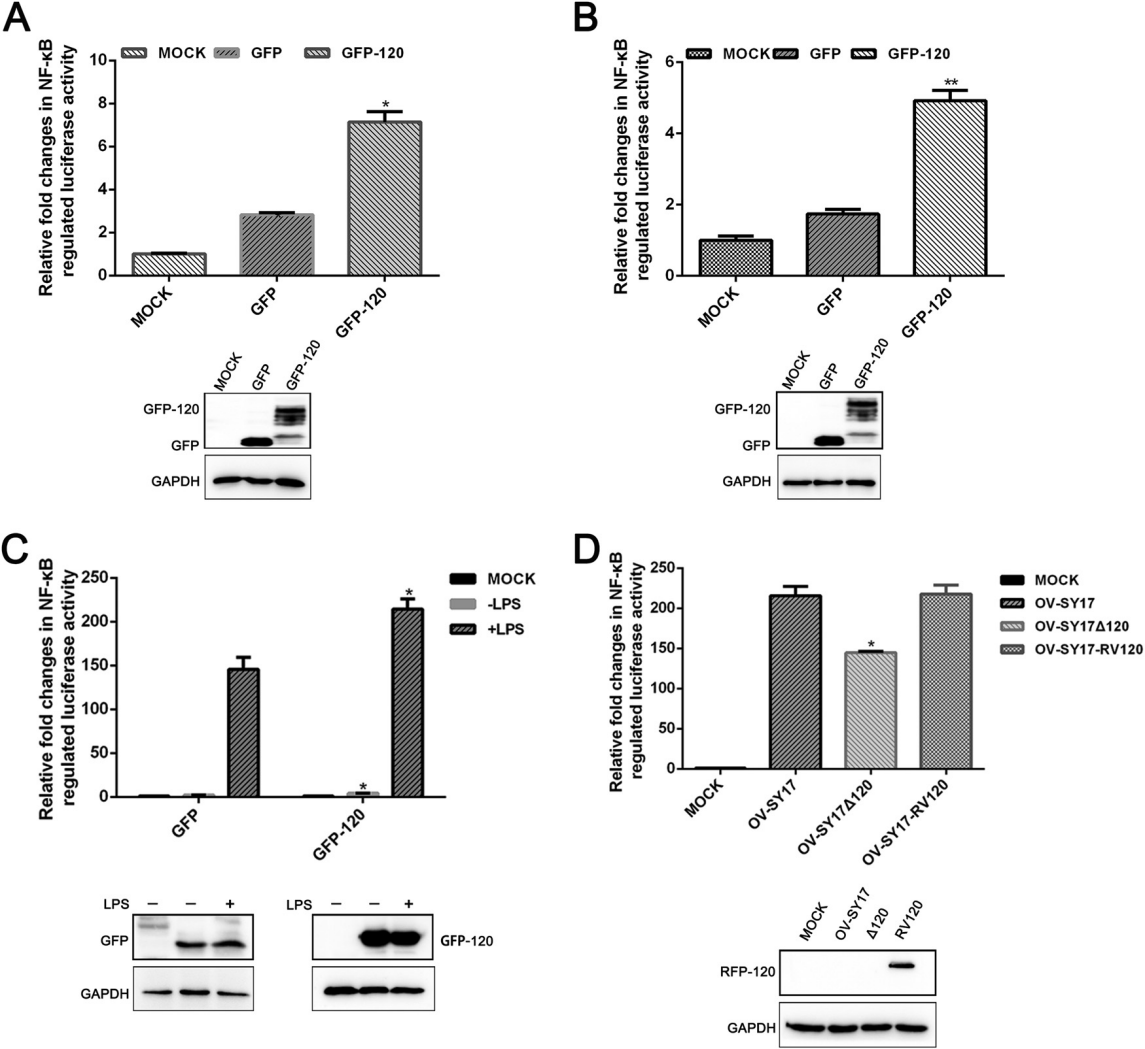
ORFV ORF120 protein is able to upregulate NF-κB activation. (A, B) OFTu cells and HeLa cells were cotransfected with pNF-κB-Luc, pRL-TK, and the eukaryotic expression vector of pIRES-puro3-EGFP120 or an empty vector using Lipofectamine 3000 in triplicate. Firefly and sea pansy luciferase activity were measured 24 h posttransfection. Statistical analysis was performed with a one-way ANOVA test followed by Dunnett’s posttest for multiple comparisons (*, *P < *0.05; **, *P* < 0.01). (C) The effect of LPS on NF-κB-regulated luciferase activity. OFTu cells were transfected with pNF-κB-Luc, pRL-TK, and the eukaryotic expression vector pIRES-puro3-GFP-ORF120 or an empty vector and subsequently treated with LPS (250 ng/ml) for 6 h. Firefly luciferase activity in OFTu cells transfected with GFP-ORF120 was represented as relative fold after normalizing to the activity in cells only transfected with GFP. Statistical significance was assessed with the unpaired two-sided *t* test using GraphPad Prism software (*, *P < *0.05). The results showed only slightly higher activity was observed in cells transfected with GFP-120 than in OFTu cells expressed GFP alone after LPS stimulation (*, *P < *0.05). (D) OFTu cells were mock infected or infected with OV-SY17, OV-SY17Δ120 ,and OV-SY17-RV120 (MOI, 10) and harvested 1 hpi. Luciferase activities were determined and normalized to uninfected cells. Statistical analysis was performed with a one-way ANOVA test followed by Dunnett’s posttest for multiple comparisons. The results are representative of three independent experiments (*, *P < *0.05; **, *P* < 0.01).

To systematically assess the involvement of the ORF120 protein in NF-κB signaling, we first examined the transcriptional activity of NF-κB in OFTu/HeLa cells overexpressing the ORF120 protein. Compared with the activity in the empty vector control cells, NF-κB-regulated luciferase activity was significantly enhanced in the OFTu cells (∼3-fold) and HeLa cells (∼2.8-fold) overexpressing the GFP-ORF120 fusion protein (Fig. 4A and B). Consistently, overexpression of the GFP-ORF120 fusion protein resulted in a remarkable increase in NF-κB luciferase activity induced by lipopolysaccharide (LPS) stimuli (Fig. 4C) compared with the activity in control GFP-expressing cells. Additionally, ORF120 deletion led to a significant reduction in NF-κB reporter activity (Fig. 4D). These observations indicated that ORF120 can act as a positive regulator of NF-κB signaling.

### ORFV ORF120 deletion suppresses the NF-κB signaling activation induced by OV-SY17 in the early phase of infection.

**FIG 5:**
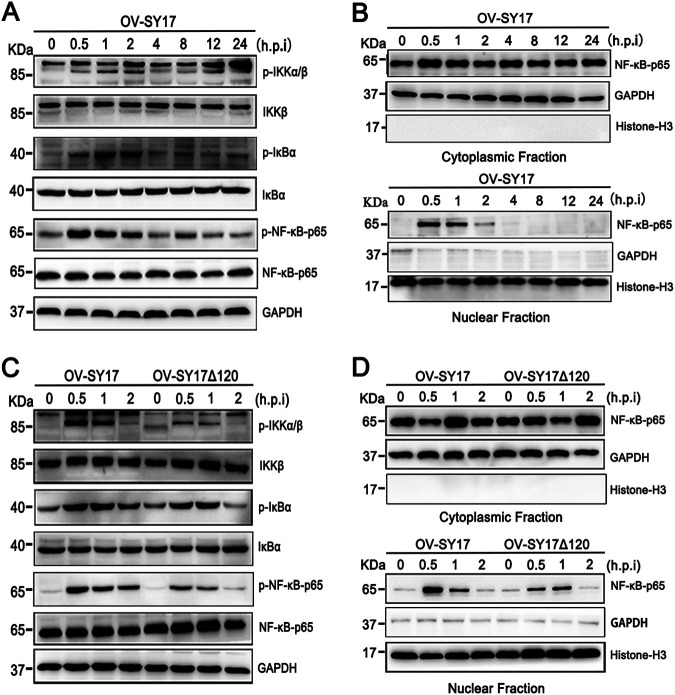
ORFV ORF120 deletion inhibits the NF-κB signaling activation induced by OV-SY17 in the early phase of infection. (A) Effect of OV-SY17 infection on the activation of NF-κB. Briefly, OFTu cells were mock infected or infected with OV-SY17 (MOI, 10) and harvested at 0.5, 1, 2, 4, 8, 12, and 24 hpi. Whole-cell extracts (50 μg) were resolved by SDS-PAGE, transferred to nitrocellulose membranes, and probed with antibodies against phosphorylated and total IKKα/β, IκBα, NF-κB-p65, and GAPDH. (B) OFTu cells were mock infected or infected with OV-SY17 (MOI, 10) and harvested at different times after infection. Cytoplasmic and nuclear fractions were extracted and resolved by SDS-PAGE, transferred, and probed with antibodies against NF-κB-p65, GAPDH, or histone H3. (C) OFTu cells were mock infected or infected with OV-SY17 and OV-SY17Δ120 (MOI, 10) and harvested at 0.5, 1, and 2 hpi. Whole-cell extracts (50 μg) were resolved by SDS-PAGE, transferred to nitrocellulose membranes, and probed with antibodies against phosphorylated and total IKKα/β, IκBα, NF-κB-p65, and GAPDH. (D) OFTu cells were mock infected or infected with OV-SY17 and OV-SY17Δ120 (MOI, 10) and harvested 0.5, 1, and 2 hpi. Cytoplasmic and nuclear fractions were extracted and resolved by SDS-PAGE, transferred, and probed with antibodies against NF-κB-p65, GAPDH, or histone H3.

To assess whether OV-SY17 mediated NF-κB activation, the phosphorylation of several signaling intermediates, including IKKα/β (Ser176/180), IκBα (Ser32/36), and NF-κB-p65 (Ser536), was analyzed by Western blotting. The results showed that the phosphorylation levels of IKKα/β, IκBα, and NF-κB-p65 were increased in the early phase of OV-SY17 infection and then decreased after 2 hpi (Fig. 5A). The densitometric analysis showed that the relative fold increases in the phosphorylation levels of these proteins in the OV-SY17-infected cells compared with the levels in the mock control cells at 0.5 hpi and 1 hpi were ∼3.1- and 4.1-fold for IKKα/β, ∼2- and 3.1-fold for IκBα, and ∼3.8- and 3.2-fold for NF-κB-p65 (data not shown), respectively. Moreover, the time kinetics of the nuclear translocation of NF-κB-p65 were studied in OV-SY17-infected OFTu cells. NF-κB-p65 was found to translocate from the cytoplasm to the nucleus during the first 30 min of infection, and this process was significantly reduced 2 hpi (Fig. 5B), which indicated that OV-SY17 regulated NF-κB in a biphasic manner in the infected cells during different phases of the viral life cycle.

To confirm that activated NF-κB was related to ORF120, we sought to determine whether the deletion of ORF120 influences the activation of NF-κB in OFTu cells. In brief, OFTu cells were mock infected or infected with OV-SY17 and OV-SY17Δ120 and harvested at 0, 0.5, 1, and 2 hpi. As shown in Fig. 5C, the phosphorylation levels of IKKα/β (Ser176/180), IκBα (Ser32/36), and NF-κB-p65 (Ser536) were reduced in the OV-SY17Δ120-infected cells compared with those of OV-SY17-infected cells. Densitometric analysis showed that the relative fold decreases in the phosphorylation levels of these proteins in the OV-SY17Δ120-infected cells compared with the levels in the OV-SY17-infected group 0.5 hpi and 1 hpi were ∼3.1- and 2.2-fold for IKKα/β, ∼1.4- and 1.3-fold for IκBα, and ∼1.4- and 1.2-fold for NF-κB-p65 (data not shown), respectively. Additionally, NF-κB-p65 was detected in the cytoplasmic and nuclear extracts by Western blotting using an anti-p65 antibody (Fig. 5D). Further densitometric analysis showed a significant decrease in translocation of NF-κB-p65 in the OV-SY17Δ120-infected OFTu cells 30 min postinfection (data not shown), which suggested that ORF120 deletion can inhibit the NF-κB signaling activation induced by the wild-type virus OV-SY17 in the early phase of infection.

**FIG 6:**
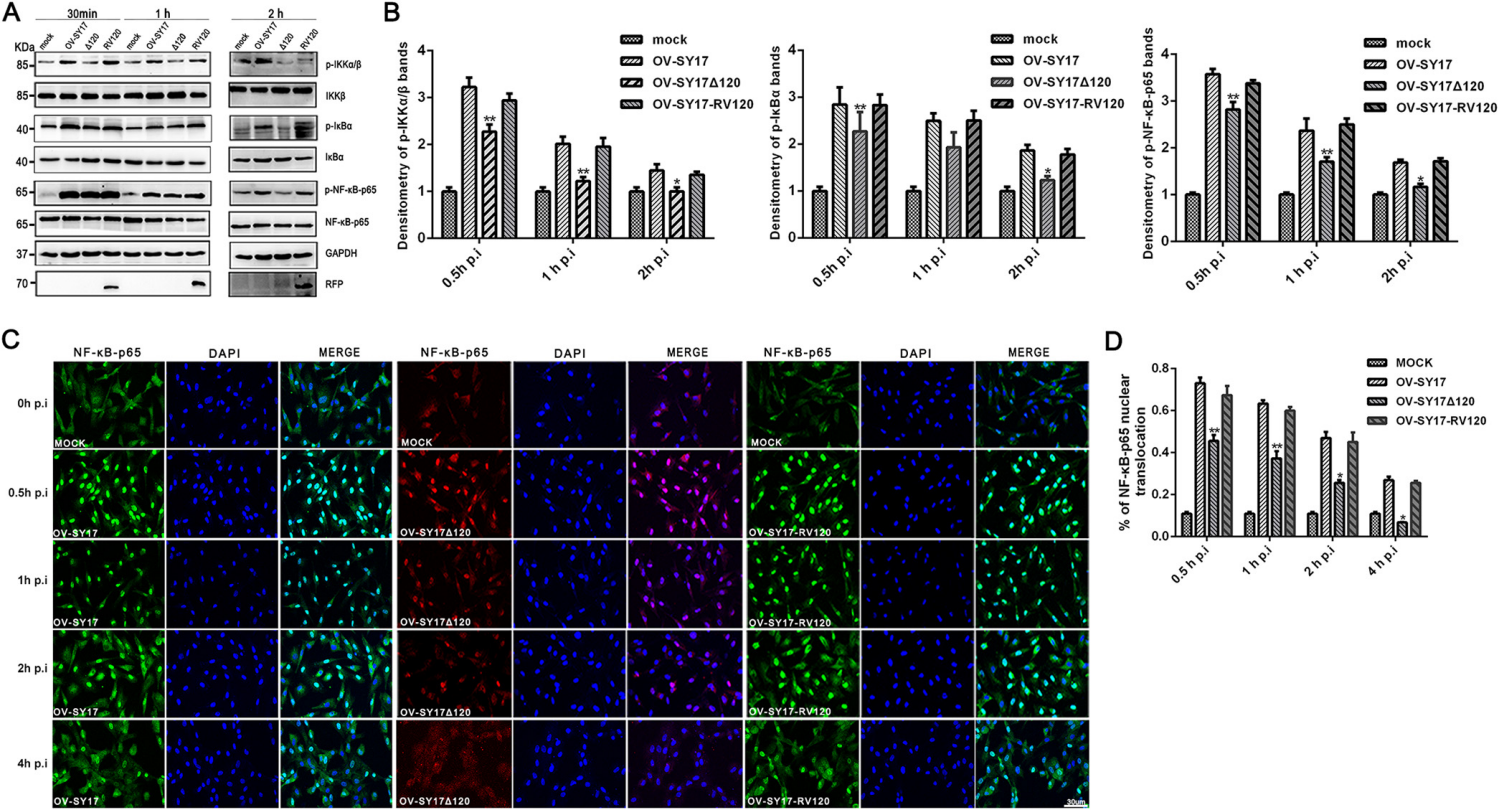
ORFV ORF120 activates the NF-κB signaling induced by OV-SY17 in the early phase of infection. (A) OFTu cells were infected with OV-SY17, OV-SY17Δ120, and OV-SY17-RV120 (MOI, 10) or mock infected and harvested at 0.5, 1, and 2 hpi. Whole-cell extracts (50 μg) were resolved by SDS-PAGE, transferred to nitrocellulose membranes, and probed with antibodies against phosphorylated and total IKKα/β, IκBα, and NF-κB-p65 and GAPDH. (B) Bands of the p-IKKα/β, p-IκBα, and p-NF-κB-p65 expression from A were analyzed by scanning densitometry and normalized to the amount of loading control, GAPDH. The fold changes are shown relative to the levels of the mock-infected cells. The data are presented as the mean values of three independent experiments (*, *P* < 0.05 and **, *P* < 0.01). (C) The effect of ORF120 on NF-κB-p65 nuclear translocation. The OFTu cells were mock infected or infected with OV-SY17, OV-SY17Δ120, or OV-SY17-RV120. The cells were fixed at the indicated times and incubated with an antibody against NF-κB-p65. The cells were then stained with Alexa Fluor 488- or 594-labeled secondary antibodies and DAPI and examined by confocal microscopy. Green/red, NF-κB p65; blue, DAPI. (D) The percentage of cells exhibiting NF-κB-p65 nuclear translocation were calculated at the indicated time points. Results are shown as mean ± SD (*n* = 400 cells/slide) for 3 independent experiments.

Consistent with the effect of the wild-type OV-SY17 virus on NF-κB activation, the revertant OV-SY17-RV120 virus was found to have the ability to compensate for the low levels of NF-κB-related protein phosphorylation, namely, for IKKα/β, IκBα, and NF-κB-p65, and to activate the NF-κB signaling pathway in the early phase of infection (Fig. 6A). Densitometric analysis showed that the phosphorylation levels of IKKα/β, IκBα, and NF-κB-p65 in OV-SY17-RV120-infected cells returned to normal levels compared with those in the OV-SY17Δ120-infected group, a finding similar to the that obtained for the wild-type virus OV-SY17-infected cells (Fig. 6B). Because the activated NF-κB subunits were translocated from the cytoplasm to the nucleus, the nuclear localization of NF-κB was further assessed by immunofluorescence. The observation of immunofluorescence indicated that the number of OV-SY17- and OV-SY-RV120-infected cells with NF-κB-p65 in the nucleus and cytoplasm was increased compared with the number of OV-SY17Δ120-infected cells (Fig. 6C). In addition, the percentage of NF-κB-p65 nuclear translocation in OFTu cells infected with OV-SY17Δ120 for 4 h was dramatically decreased compared with that of the OV-SY17- and OV-SY-RV120-infection groups (Fig. 6D), which ruled out the possibility that the mild phenotype caused by the deletion of ORF120 is a simply delayed activation of NF-κB signaling. These results indicated that ORFV ORF120 activated NF-κB signaling in the early phase of infection.

### Overexpression of ORFV ORF120 transiently activates the NF-κB pathway and promotes NF-κB-p65 nuclear translocation.

**FIG 7:**
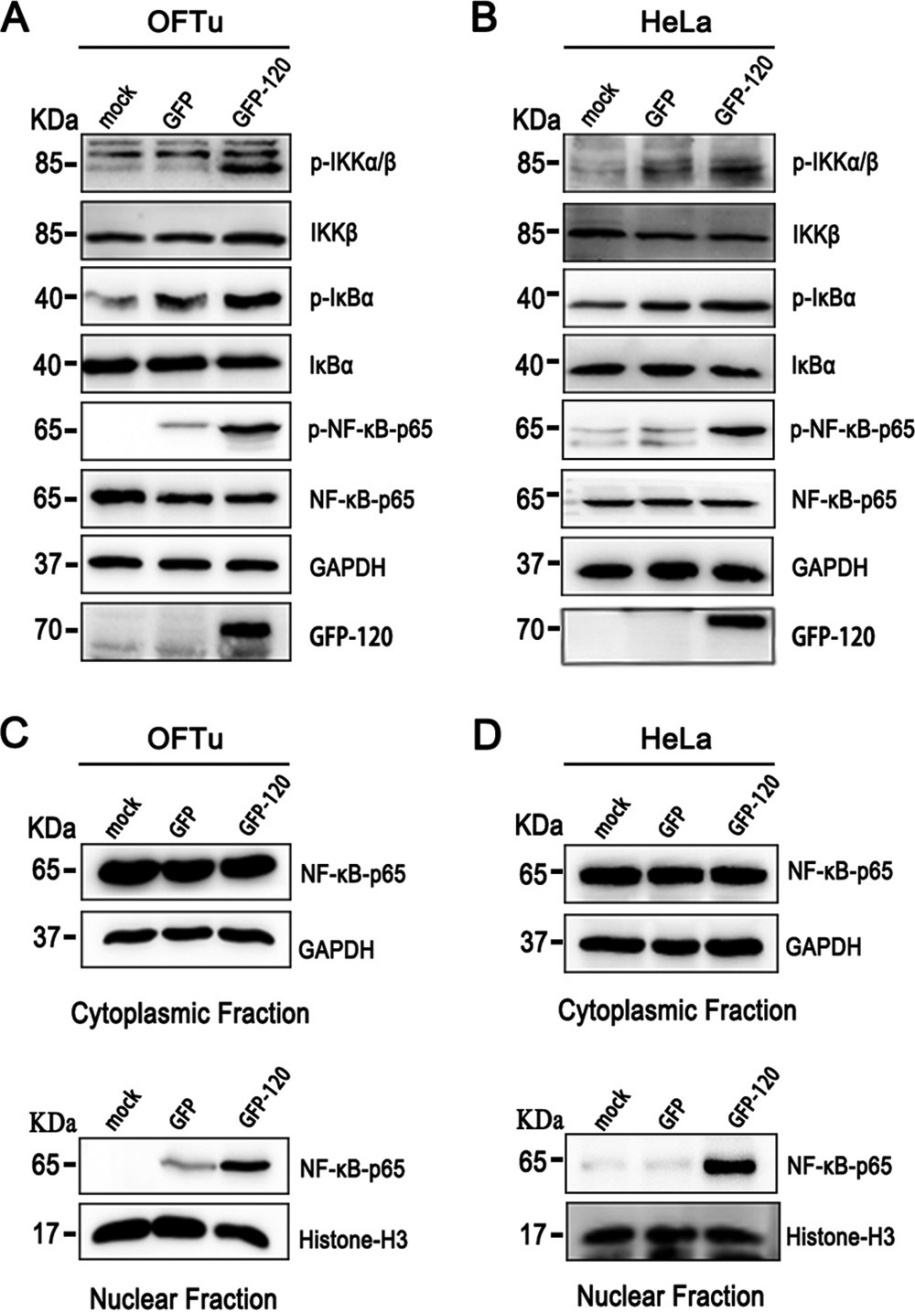
Overexpression of ORFV ORF120 transiently activates the NF-κB pathway and NF-κB-p65 nuclear translocation. (A, B) OFTu and HeLa cells were transiently transfected with plasmids encoding GFP-120 (pIRES-puro3-EGFP120) or GFP control (pIRES-puro3-EGFP) and incubated for 24 h. Whole-cell extracts (50 μg) were resolved by SDS-PAGE, transferred to nitrocellulose membranes, and probed with antibodies against phosphorylated and total IKKα/β, IκBα, and NF-κB-p65 and GAPDH. (C, D) Cytoplasmic and nuclear fractions were extracted and resolved by SDS-PAGE, transferred, and probed with antibodies against NF-κB-p65, GAPDH, or histone H3. The results are representative of three independent experiments.

To confirm the effect of ORFV ORF120 on the activation of NF-κB signaling, OFTu and HeLa cells were transiently transfected with plasmids encoding GFP-120 (pIRES-puro3-EGFP120) or GFP control (pIRES-puro3-EGFP). The results of the Western blot analysis showed that the GFP-120 fusion protein increased the phosphorylation levels of IKKα/β, IκBα, and NF-κB-p65 (Fig. 7A and B). However, the difference in the phosphorylation levels of IKKα/β, IκBα, and NF-κB-p65 was observed in the transfected OFTu cells and HeLa cells, which might be responsible for the different cell types. Additionally, similar levels of NF-κB-p65 nuclear translocation were detected in the OFTu cells and HeLa cells expressing the GFP-120 fusion protein (Fig. 7C and D). In summary, ORFV ORF120 activated the NF-κB signaling pathway.

### The ORFV ORF120 protein interacts with G3BP1.

**FIG 8:**
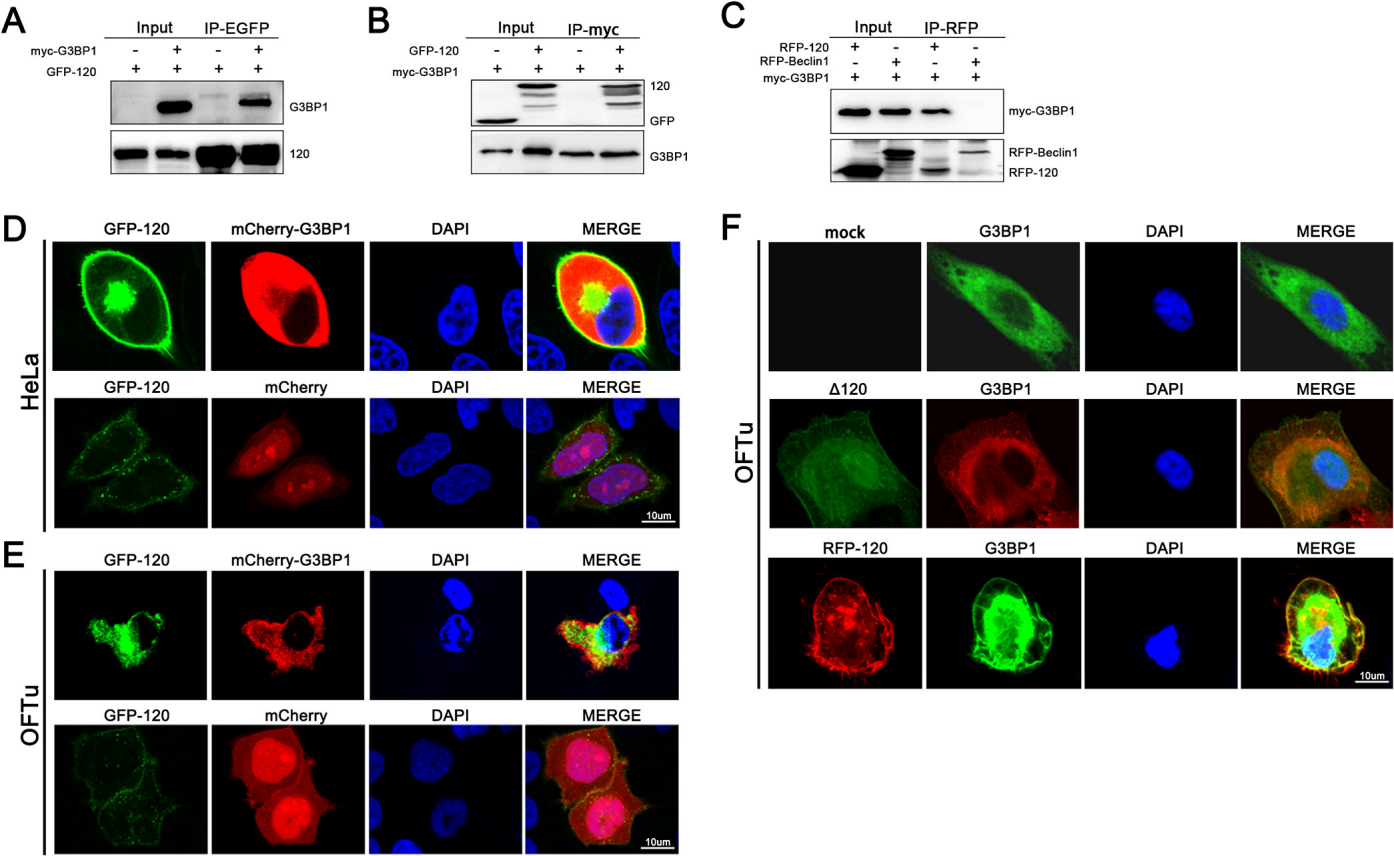
ORFV ORF120 interacts with G3BP1. (A, B) OFTu cells were cotransfected with pIRES-puro3-EGFP120 and pCMV-N-myc-G3BP1 or either empty vector and harvested. The protein extracts were immunoprecipitated with antibodies and examined by Western blotting using anti-GFP or anti-myc antibodies. (C) OFTu cells were cotransfected with myc-G3BP1 and pcDNA3.1-RFP-Becline1 and harvested at 24 h posttransfection. Protein extracts of the transfected cells were immunoprecipitated with rabbit anti-RFP antibody. OFTu cells were transfected with myc-G3BP1 and then infected with RFP-tagged ORF120 revertant virus at 24 h posttransfection. Then, cells were harvested and immunoprecipitated with rabbit anti-RFP antibody. (D, E) HeLa cells and OFTu cells were cotransfected with pIRES-puro3-EGFP120 and pcDNA3.1-mCherry-G3BP1 or empty vectors and, 24 h later, fixed with 4% formaldehyde, stained with DAPI, and examined by confocal microscopy. (F) OFTu cells were mock infected or infected with OV-SY17Δ120 and OV-SY17-RV120 (MOI, 10). The samples were fixed for 15 min with 4% paraformaldehyde at RT and then incubated with primary antibody against G3BP1 and the appropriate Alexa Fluor 488- or 594-labeled secondary antibodies as described above. Green/red, EGFP/G3BP1; blue, DAPI.

The DUAL membrane yeast two-hybrid system indicated that G3BP1 was an interactor of the ORFV ORF120 protein (data not shown), which has been confirmed to participate in the host antiviral response, based on various strategies, such as stimulating the activation of innate immune transcriptional responses through NF-κB (39). To confirm that the ORF120 protein interacts with G3BP1, coimmunoprecipitation assays were performed using protein extracts from OFTu cells transfected with pIRES-puro3-EGFP120, pCMV-N-myc-G3BP1, or empty vector. The immunoprecipitates were detected by Western blotting using anti-GFP or anti-myc antibodies. The ORF120 protein was found to coimmunoprecipitate with G3BP1 (Fig. 8A and B). Additionally, an immunoprecipitation assay performed for G3BP1 during RFP-tagged ORF120 revertant virus infection also showed that G3BP1 could interact with RFP-tagged ORF120 revertant virus using anti-myc or anti-RFP antibodies (Fig. 8C). Furthermore, the interaction of the ORF120 protein with G3BP1 was confirmed by confocal microscopy analysis. Immunofluorescence analysis showed that the GFP-ORF120 fusion protein colocalized with G3BP1 in HeLa cells (Fig. 8D) and OFTu cells (Fig. 8E). In addition, we found that the RFP-120 protein colocalized with G3BP1 in the OFTu cells infected with OV-SY17-RV120; however, no colocalization was observed in the OV-SY17Δ120-infected cells (Fig. 8F).

### The identification of the binding domain of G3BP1.

**FIG 9:**
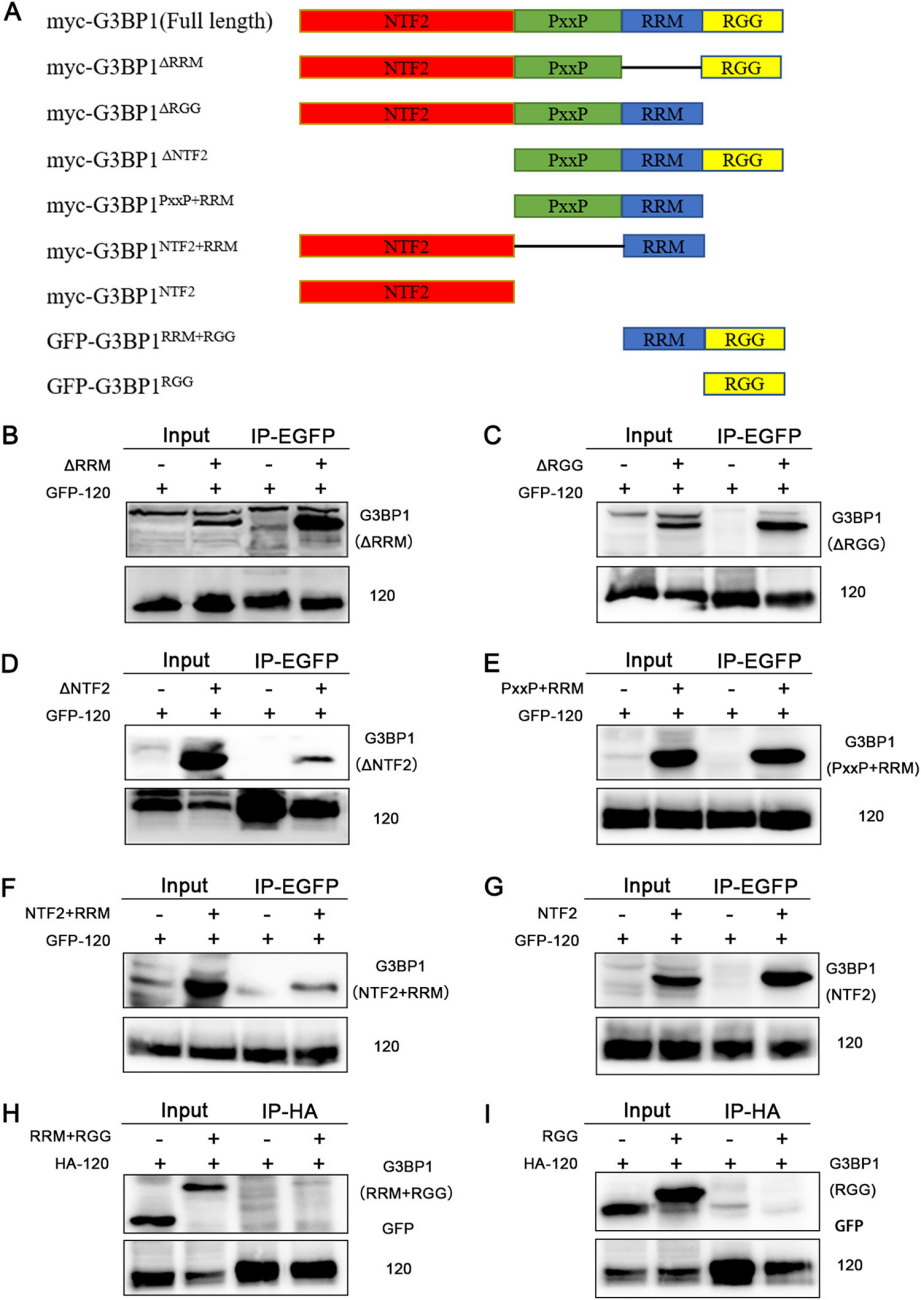
Identification of the potential ORF120-G3BP1 binding sites. (A) The truncated forms of G3BP1 utilized in B to I. (B to I) The potential ORF120-G3BP1 binding sites were analyzed by immunoprecipitation with HEK293T cells overexpressing EGFP-120/HA-120 together with the GFP-truncated G3BP1.

G3BP1 is a multidomain protein with an N-terminal nuclear transport factor 2-like domain (NTF2 domain), a PxxP domain, an RNA recognition motif (RRM domain), and an arginine-glycine-glycine motif (RGG domain). To identify the potential ORF120-G3BP1 binding sites, several plasmids expressing truncated forms of G3BP1 were constructed (Fig. 9A). Immunoprecipitation with HEK293T cells overexpressing EGFP-ORF120 or HA-ORF120 together with myc-tagged truncated G3BP1 was performed using the GFP or HA antibody. The results indicated that the myc-G3BP1^ΔRRM^ mutant (Fig. 9B), myc-G3BP1^ΔRGG^ (Fig. 9C), and myc-G3BP1^ΔNTF2^ mutant (Fig. 9D) interacted with the ORF120 protein. In addition, we found that the myc-G3BP1^PxxP+RRM^ mutant (Fig. 9E), myc-G3BP1^NTF2+RRM^ mutant (Fig. 9F), and myc-G3BP1^NTF2^ mutant (Fig. 9G) also bound the ORF120 protein. However, GFP-G3BP1^RRM+RGG^ (Fig. 9H) and GFP-G3BP1^RGG^ (Fig. 9I) could not interact with the ORF120 protein. Therefore, the NTF2 and PxxP domains of G3BP1 were determined to be the key ORF120-binding sites.

### G3BP1 is involved in NF-κB activation induced by the ORFV ORF120 protein.

**FIG 10:**
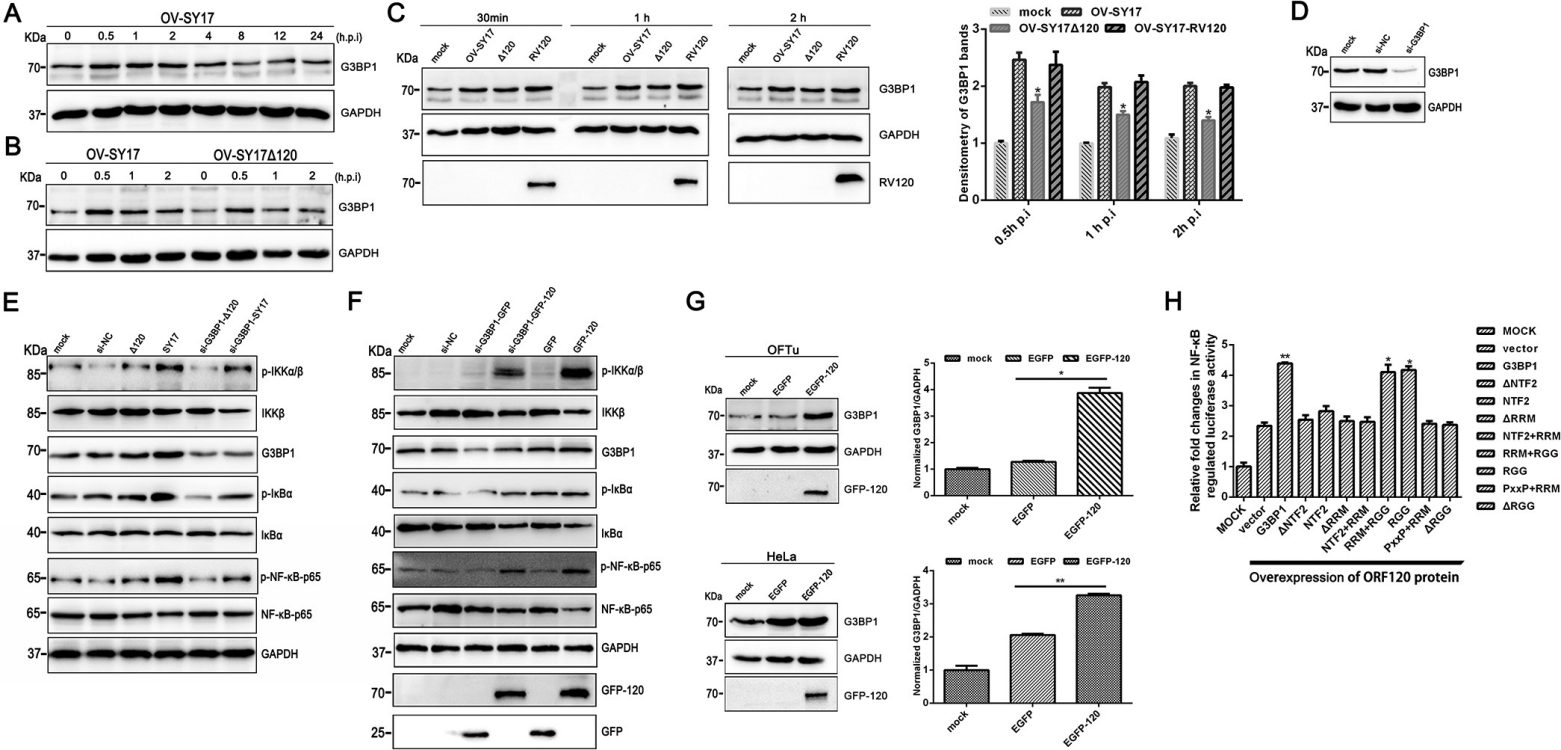
G3BP1 is involved in NF-κB activation induced by the ORFV ORF120 protein. (A) The changes of G3BP1 expression levels were detected in OV-SY17-infected cells. OFTu cells were mock infected or infected with OV-SY17 (MOI, 10) and harvested at 0.5, 1, 2, 4, 8, 12, and 24 hpi for Western blot analysis. The blots were incubated with primary antibodies against G3BP1 and GAPDH. The blots were developed using a chemiluminescence assay. (B) The effect of the ORF120 protein on host factor G3BP1 expression levels. OFTu cells were mock infected or infected with OV-SY17 and OV-SY17Δ120, respectively, for 0.5, 1, and 2 h and harvested for Western blot analysis. The blots were incubated with primary antibodies against G3BP1 and GAPDH. The blots were developed using a chemiluminescence assay. (C) OFTu cells were mock infected or infected with OV-SY17, OV-SY17Δ120, or OV-SY17-RV120 (MOI, 10) and harvested at 0.5, 1, and 2 hpi. Then, the cell samples were subjected to Western blot analysis. The blots were incubated with primary antibodies against G3BP1 and GAPDH. The blots were developed using a chemiluminescence assay. Statistical significance was assessed with the unpaired two-sided *t* test using GraphPad Prism software (*, *P < *0.05). (D) The silencing effect of siRNA targeting G3BP1 was detected by Western blotting. (E) OFTu cells were transduced with G3BP1-specific siRNA for 48 h. Then, cells were infected with OV-SY17 or OV-SY17Δ120 (MOI, 10) and harvested at 0.5 hpi. Whole-cell extracts (50 μg) were resolved by SDS-PAGE, transferred to nitrocellulose membranes, and probed with antibodies against G3BP1, GAPDH, and phosphorylated and total IKKα/β, IκBα, and NF-κB-p65. (F) OFTu cells were transduced with G3BP1-specific siRNA for 48 h. Then, cells were transfected with plasmids encoding GFP control or GFP-120 and incubated for 24 h. Whole-cell extracts (50 μg) were resolved by SDS-PAGE, transferred to nitrocellulose membranes, and probed with antibodies against G3BP1, GAPDH, and phosphorylated and total IKKα/β, IκBα, and NF-κB-p65. (G) OFTu and HeLa cells were transfected with pIRES-puro3-EGFP120 and pIRES-puro3-EGFP plasmids, respectively. Twenty-four hours posttransfection, the cell samples were harvested and subjected to Western blot analysis. The blots were incubated with primary antibodies against G3BP1 and GAPDH. The blots were developed using a chemiluminescence assay. Further densitometry data were normalized by the amount of GAPDH. The fold changes are shown relative to the levels of the GFP control group. Statistical analysis was performed with a one-way ANOVA test followed by Dunnett’s posttest for multiple comparisons. The data are presented as the mean values of three independent experiments (*, *P < *0.05; **, *P* < 0.01). (H) The effect of full-length G3BP1 and truncated G3BP1 on the transcription activity of NF-κB induced by ORF120 was investigated by dual-luciferase reporter assay. Briefly, HEK293T cells were seeded into 12-well plates and then cotransfected with NF-κB luciferase reporter pNF-κB-Luc, *Renilla* luciferase encoding plasmid pRL-TK, eukaryotic expression vector pIRES-puro3-EGFP120, and mutants of G3BP1 or empty vector using Lipofectamine 3000 in triplicate. Twenty-four hours after transfection, the luciferase activity was measured using the dual-luciferase reporter assay system according to the manufacturer’s instructions. Firefly luciferase activity was normalized to *Renilla* luciferase activity. Statistical analysis was performed with the unpaired two-sided *t* test using GraphPad Prism software (*, *P < *0.05; **, *P* < 0.01).

To determine whether or not the ORFV ORF120-mediated NF-κB activation involves G3BP1, the effect of the ORF120 protein on G3BP1 expression levels during viral infection was explored. As shown in Fig. 10A, the expression levels of G3BP1 were increased sharply at first and then declined in OV-SY17-infected cells. A comparison of the degree of G3BP1 expression of the OV-SY17-infected cells and OV-SY17Δ120-infected cells revealed that the expression levels of the G3BP1 protein were decreased in the OV-SY17Δ120-infected cells (Fig. 10B), which was partially reversed after infection with OV-SY17-RV120 (Fig. 10C). Furthermore, OFTu cells were transduced with G3BP1-specific small interfering RNA (siRNA) resulting in 85% knockdown of G3BP1 (Fig. 10D) and then infected with OV-SY17 and OV-SY17Δ120, respectively. As shown in Fig. 10E, the phosphorylation levels of IKKα/β, IκBα, and NF-κB-p65 in siRNA-G3BP1 cells infected with OV-SY17 and OV-SY17Δ120 were lower than those in the control cells (not treated with siRNA-G3BP1), which indicated that G3BP1 was involved in NF-κB activation induced by the ORF120 protein. However, only slight differences in the phosphorylation levels of IKKα/β, IκBα, and NF-κB-p65 were observed in OFTu cells that overexpressed the GFP-120 protein and GFP-120 plus G3BP1-specific siRNA (Fig. 10F), which might be due to overexpression of the GFP-120 protein in siRNA-G3BP1-treated cells temporarily contributing to the expression levels of G3BP1 to some extent. Additionally, we observed an increase in G3BP1 expression in ORF120-overexpressed OFTu cells and HeLa cells (Fig. 10G), which suggested that the degree of G3BP1 expression was correlated with ORF120 expression. A dual-luciferase reporter assay further revealed that ORF120 could positively regulate the NF-κB pathway through the full-length G3BP1 or the domain of G3BP1^RRM+RGG^ (Fig. 10H).

### ORFV ORF120 gene promotes virus virulence in the natural host.

**FIG 11:**
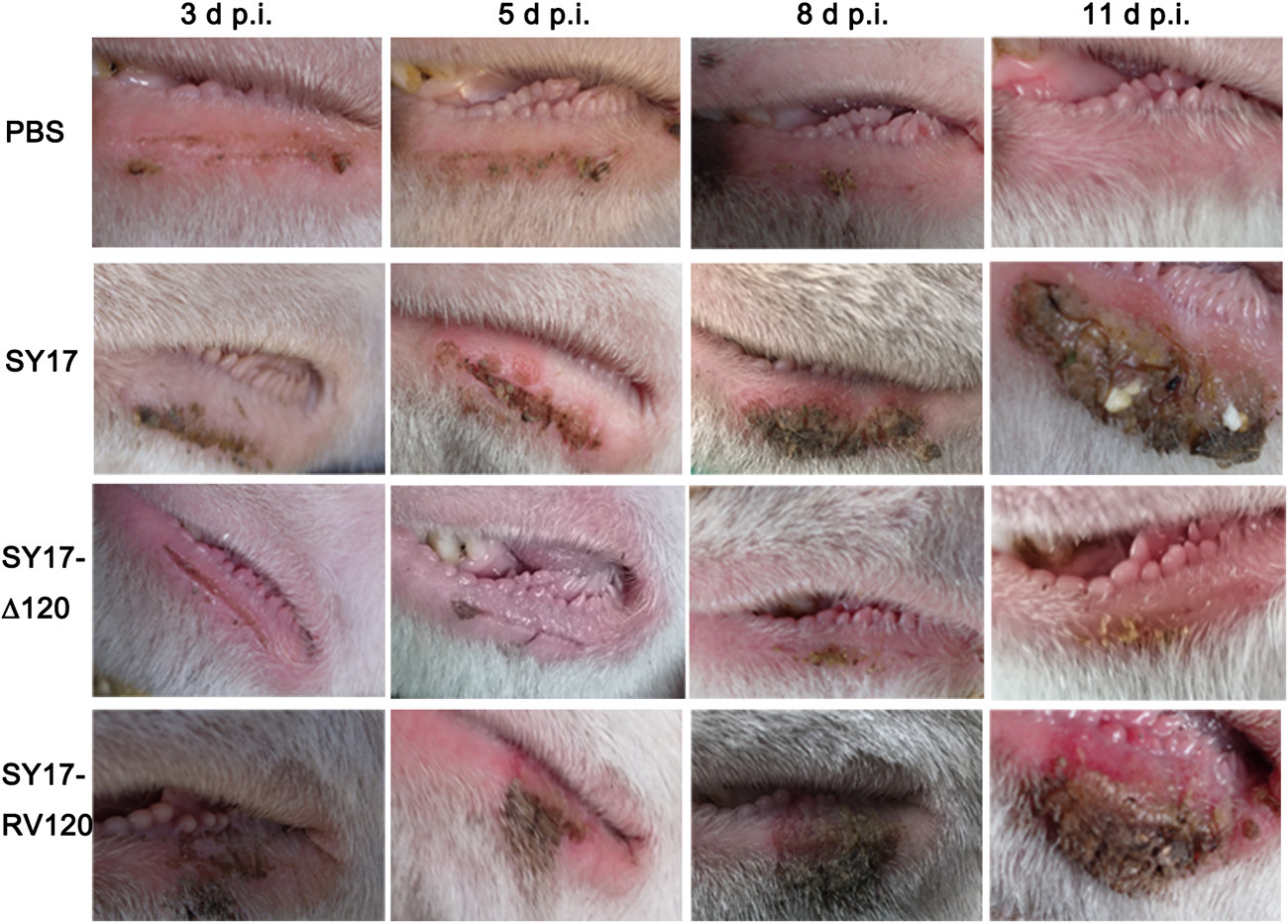
The ORFV ORF120 gene promotes virus virulence and pathogenesis in the natural host. Sixteen 4- to 5-month-old lambs were randomly divided into 4 groups (*n* = 4 per group), including 1 control group and 3 experimental groups. Under general anesthesia, the infected groups were inoculated with OV-SY17, OV-SY17Δ120, or OV-SY17-RV120 by scarification with virus inoculum containing 10^7.37^ TCID_50_/ml in the inferior lip, respectively. Lambs in the control group were inoculated with PBS in the same manner. Then, the inoculated animals were monitored daily for at least 20 days. The characteristic clinical signs and Orf lesions of all of the groups were observed and recorded daily.

To determine whether ORF120 is essential for virus virulence in the natural host, lambs were inoculated with phosphate-buffered saline (PBS) (*n* = 4), OV-SY17 (*n* = 4), OV-SY17Δ120 (*n* = 4), or OV-SY17-RV120 (*n* = 4) by scarification with virus inoculum containing 10^7.37^ 50% tissue culture infective dose (TCID_50_)/ml in the inferior lip and monitored daily for 20 days. The characteristic Orf lesions were observed in the wild-type parent virus OV-SY17-inoculated group and the revertant virus OV-SY17-RV120-inoculated group as early as 3 days postinoculation. The vesicles, pustules, and ulcerated lesions subsequently developed until covered with the degenerated scabs at 18 days postinoculation. In contrast, the lambs that were inoculated with OV-SY17Δ120 displayed the significantly less severe lesions (Fig. 11). The above results indicated that the ORF120 gene plays a critical role in virulence and pathogenesis.

## DISCUSSION

Various viruses have acquired sophisticated mechanisms to regulate the NF-κB signaling pathways to counteract antiviral responses, thus favoring their replications. Similar to other poxviruses, ORFV carries several viral proteins, including open reading frames (ORFs) 002, 024, 073, 119, and 121, that effectively block the induction of NF-κB activation to contribute to viral replication (29–33). However, many viruses have been demonstrated to optimize viral replication by relying on the activation of NF-κB. For example, the herpesvirus (HSV) structural protein UL37 can activate NF-κB through the TRAF6 adaptor protein, and NF-κB activity is necessary for efficient viral replication (42). ASFV IAP-like protein (A224L) is also able to activate NF-κB, thus inhibiting apoptotic cell death by blocking caspase-3 activity to benefit viral survival (43). Respiratory syncytial virus (RSV) M2-1 protein was confirmed to be sufficient to activate NF-κB, and the activation of NF-κB depended on viral replication in the infected cells (44).

The present study explored, for the first time, the role of the ORFV ORF120 gene, for which the function was unknown, located in the right-terminal genomic fragment, by using a OV-SY17Δ120 deletion mutant. A decrease in the expression levels of NF-κB-mediated proinflammatory cytokines was observed in the OV-SY17Δ120-infected cells, as indicated by RNA-seq analysis and qPCR. Additionally, a remarkable decrease in NF-κB-regulated luciferase activity was detected in the OV-SY17Δ120-infected cells, while a significant increase was observed when the ORF120 protein was overexpressed. The signal transduction pathway(s) mediated by ORF120 to activate NF-κB was confirmed by inducing the phosphorylation of the IKK complex subunits IKKα/β, phosphorylation and degradation of IκBα, and phosphorylation and nuclear translocation of NF-κB p65 in the early phase of infection. As shown in Fig. 5, OV-SY17 can activate NF-κB in the early phase of infection, probably because of the inducing effect of the ORF120 protein, which may be critical in part for this effect. However, even in the absence of ORF120, the OV-SY17Δ120 mutant still retained the ability to promote NF-κB activation, albeit at a less degree than that by the wild-type virus (Fig. 5C). Thus, some additional viral proteins carried by ORFV may also promote the transient activation of the NF-κB pathway. Taken together, our results confirm that ORFV ORF120 protein expression is sufficient to activate NF-κB signaling, which implies that ORFV may regulate NF-κB through a biphasic mechanism. Thus, taking into account the importance of transient activation of NF-κB during the early phase of infection will help us to fully understand the unique feature of ORFV infection and pathogenesis.

Considering that the ORF120 protein has the ability to activate NF-κB, its ability to induce NF-κB represents a possible function. To further explore the potential role of the ORF120 protein, the transcription kinetics and intracellular localization in infected cells were investigated. The transcription of ORF120 showed similar kinetics to those of ORFV127 (an early viral gene) and was slightly decreased by the presence of AraC, suggesting that ORF120 is an early-late gene in the viral cycle. Although ORF120 was clearly expressed throughout the course of infection (Fig. 2A), it promoted NF-κB activation only in the early phase of infection. The presence of viral NF-κB inhibitors (ORFV002, ORFV024, and ORFV121) expressed at a later time may lead to the overall silencing effect to the NF-κB pathway (29, 30, 33). The subcellular localization of the ORF120 protein was observed in the OFTu cells infected with the RFP-labeled OV-SY17-RV120 revertant mutant. As observed in Fig. 2B, the red fluorescence intensity in a spotty pattern displayed a gradual increase (from 0.5 hpi to 24 hpi) in the cytoplasm of the infected cells, whereas a very small amount of ORF120 protein was observed in the nucleus. The results were also demonstrated in the OFTu cells and HeLa cells overexpressing the GFP-ORF120 fusion protein. The presence of the ORF120 protein in the nuclei of the OV-SY17-RV120 revertant mutant-infected and GFP-ORF120-transfected cells was unexpected. Considering these results, we conclude that ORF120 may be transported into the nucleus upon binding to cytoplasmic factors. However, the lack of homology between the ORF120 protein and known poxviral proteins or any other DNA virus proteins capable of modulating the NF-κB signaling pathway prompted us to explore the mechanism by which ORF120 activates NF-κB.

G3BP1 is a key component of RNA SGs. G3BP1-induced SGs have been confirmed to be related to the activation of innate immune transcriptional responses through NF-κB and JNK (35). In addition, a recent study suggested that G3BP1 appears to be critical for the general recognition of different types of DNA by cGAS, which contributes to cGAS-mediated antiviral responses (39). In the present study, the DUAL membrane system analysis revealed a G3BP1 interaction with the ORF120 protein. The interaction of the ORF120 protein with G3BP1 was further confirmed using coimmunoprecipitation assays and colocalization observation by microscopy. Considering that G3BP1 is a multidomain protein, a series of truncated G3BP1 forms consisting only of the NTF2 domain, PxxP domain, RRM domain, or RGG domains were constructed and used for the identification of the potential ORF120-G3BP1 binding sites. Using these mutants, the NTF2 and PxxP domains of G3BP1 were determined to be the key ORF120 binding sites on G3BP1. Because the NTF2 domain is related to nuclear transport and localization, the presence of the ORF120 protein in nuclei is explained by the ORF120 protein likely binding with G3BP1.

G3BP1, an antiviral protein, can promote multiple innate antiviral immune responses, including NF-κB activation, stress granule (SG) formation, and type I interferon production. Thus, in response to ORFV infection, the expression of G3BP1 was quickly increased during the early stage of infection (as early as 30 min) to induce an antiviral innate immune response. This might be the underlying mechanism for a quick upregulation of the G3BP1 protein level at the early stage of ORFV infection. However, the level of G3BP1 quickly declined with prolongation of infection time. In a recent study, we confirmed that the interaction of ORF120 and G3BP1 could inhibit the formation of SGs and the release of IFN-γ (data not shown), implying that ORF120 might be involved in the regulation of antiviral immune responses mediated by G3BP1. Therefore, the infection with the OV-SY17Δ120 deletion mutant would promote the formation of SGs and the release of IFN-γ, which further enhanced host antiviral innate immunity. In the study, ORFV ORF120-mediated NF-κB activation involving G3BP1 was further determined by siRNA-mediated knockdown/overexpression of G3BP1. The expression levels of the G3BP1 were decreased in the OV-SY17Δ120-infected cells but reversed in the OV-SY17-RV120-infected cells. And an increase in G3BP1 expression was observed in ORF120-overexpressed OFTu cells and HeLa cells, which suggested that the degree of G3BP1 expression was correlated with ORF120 expression. Further study found that the phosphorylation levels of IKKα/β, IκBα, and NF-κB-p65 in siRNA-G3BP1 cells infected with OV-SY17 and OV-SY17Δ120 were lower than those in the control cells (not treated with siRNA-G3BP1), which indicated that G3BP1 was involved in ORF120-induced NF-κB activation to some extent. Additionally, OV-SY17 was observed to be able to activate the NF-κB pathway in siRNA-G3BP1 cells, which implied that ORF120 might be activate NF-κB pathway by other ways. Thus, one interpretation off this finding is that activation of NF-κB may be only partially dependent on G3BP1. Also, an interesting phenomenon was found; levels of total IκBα remained unchanged during infection, which might be due to the existence of other viral factors. In vaccinia virus, the A49 protein has been confirmed to prevent IκBα phosphorylation and degradation by targeting the E3 ligase β-TrCP, so it is possible that a functionally equivalent factor exists in Orf virus (45). Additionally, a dual-luciferase reporter assay further revealed that ORF120 could positively regulate the NF-κB pathway through the full-length G3BP1 or the domain of G3BP1^RRM+RGG^.

Although the details of the mechanism by which ORF120 activates NF-κB remain to be elucidated fully, from our results, we can propose the basic outline of this process. The ORFV ORF120 protein interacts with the G3BP1 NTF2 and PxxP domains and positively regulates the NF-κB pathway through the full-length G3BP1 or the domain of G3BP1^RRM+RGG^. However, the possible effect of the ORF120-G3BP1 interaction on the innate immune response and whether ORF120 could activate the NF-κB pathway by other ways remain unknown and need to be further studied.

## MATERIALS AND METHODS

### Cells and viruses.

Primary ovine fetal turbinate (OFTu) cells were cultured in minimal essential medium (MEM) (Gibco, Invitrogen) supplemented with 10% fetal bovine serum (FBS), 2 mM l-glutamine, 100 U/ml penicillin, 100 μg/ml streptomycin/ml, and 20 μg/ml nystatin at 37°C in a 5% CO_2_ incubator. HeLa cells and HEK293T cells were cultured in Dulbecco’s modified Eagle’s medium (DMEM) supplemented with 10% FBS, 2 mM l-glutamine, 100 U/ml penicillin, 100 μg/ml streptomycin, and 20 μg/ml nystatin. ORFV strain OV-SY17 (46) was used to construct a ORFV ORF120 gene deletion virus (OV-SY17Δ120) expressing enhancing green fluorescent protein (EGFP) and for experiments involving wild-type virus infection. The deletion mutant OV-SY17Δ120 was generated and used as the parental virus for the generation of a revertant virus (termed OV-SY17-RV120) inserting the red fluorescent protein (RFP) gene and using a FLAG-tag as a reporter of gene expression.

### Plasmids.

To construct the ORFV ORF120 expression plasmid, the enhanced green fluorescent protein (EGFP) was amplified using an pEGFP-N1 plasmid as a template and first cloned into a pIRES-puro3 vector (Clontech). The ORF120 fragment was ligated upstream of the EGFP coding sequence, and the resultant EGFP fusion protein (ORF120-GFP) was expressed. The HA-ORF120 plasmid was generated by subcloning the ORF120 coding sequence into a pCMV-N-HA vector (Clontech). G3BP1 was amplified by PCR and inserted into a pcDNA3.1-mCherry vector (Invitrogen) and a pCMV-N-myc vector (Clontech). The primers used for plasmid construction were listed in Table 2.

**TABLE 2:** Sequences of the oligonucleotide primers used in the study

Primer	Sequence
pEGFP-Fw	CCGGAATTCATGGTGAGCAAGGGCGAG
pEGFP-Rv	AAGGAAAAAAGCGGCCGCTTACTTGTACAGCT
pORF120-EGFP-Fw	CTAGCTAGCATGCGTCTAATCTTAGCG
pORF120-EGFP-Rv	CCATGAATTCTCTGGGGTCCGCGCGC
mCherry-G3BP1-Fw	CTAGCTAGCGCCACCATGGTGATGGAGAAGCCTAG
mCherry-G3BP1-Rv	CCCAAGCTTCTGCCGTGGCGCAAGCCCCCTTC
HA-120-F	CCGGAATTCGGATGCGTCTAATCTTAGCGC
HA-120-R	CGGGGTACCTTATCTGGGGTCCGCGCGC
myc-G3BP1-Fw	CCGGAATTCGGGTGATGGAGAAGCCTAGTC
myc-G3BP1-Rv	GGAAGATCTTCACTGCCGTGGCGCAAGC
ORF120 LF-Fw	CCCAAGCTTAGTACTTCGAGGTCTGCG
ORF120 LF-Rv	ACGCGTCGACACGTGTTTGGAGTGCTTG
ORF120 RF-Fw	TCTTATGCGGCCGCAGAGCTCAAGGACTACCTCG
ORF120 RF-Rv	ATTCGCAGATCTTCAGGCAGTCGTTCATGGAC
HA-ORF120-Fw	CCGGAATTCGGATGCGTCTAATCTT AGCGC
HA-ORF120-Rv	CGGGGTACCTTATCTGGGGTCCGCGCGC
ORF120 Fw	GCTCGACGAGTTCGGAACC
ORF120 Rv	GCAGTCACAGAGTCCCTG
ORFV055 Fw	CGCCCGACGAGGAGATTTACG
ORFV055 Rv	GGTTCAGGTATTTCGCCTGGAAGC
ORFV127 Fw	CTCCTCGACGACTTCAAAGG
ORFV127 Rv	TATGTCGAACTCGCTCATGG
ORF119 Fw	TGAGCCCCTCCATCTTCGG
ORF119 Rv	AATCGCTGTCGCTGTCGC
ORFV121 Fw	AAGACATTGCTCGCCACTCG
ORFV121 Rv	ACAGAACTTCCTCCACTTTGC
IL1β-F	AAATCCCTGGTGCTGGATAG
IL1β-R	GTTGTCTCTTTCCTCTCCTTGT
IL-8-F	CTTCCAAGCTGGCTGTTG
IL-8-R	ATTTGGGGTGGAAAGGTG
ICAM1-F	CGGAGTCACCCGTGAAGTGG
ICAM1-R	TGCCCAGAGTGCCCAAGATG
NFKBIA-F	TCCGCCAAGTGAAGGGAGAC
NFKBIA-R	GCTCACAGGCAAGGTGTAGGG
PTGS2-F	GGAGGTCTTTGGTCTGGTGC
PTGS2-R	CCTATCAGGATTAGCCTACTTG

### Construction and characterization of the ORFV ORF120-deletion mutant and RFP-tagged ORF120 revertant viruses.

A recombination cassette containing a reporter gene (EGFP) preceded by the early-late vaccinia virus (VACV) VV7.5 promoter flanked by ORF120 left (819 bp) and right (852 bp) flanking regions (LFs and RFs, respectively) was generated by PCR using the OV-SY17 genome as a template and then cloned into a pUC57-EGFP transfer vector. The primers used for ORFV ORF120 left and right flanking regions were listed in Table 2. The OV-SY17Δ120 deletion mutant was created via homologous recombination between the parental OV-SY17 and the recombination transfer vector pUC57-120LF-EGFP-120RF. Briefly, OFTu cells cultured in 6-well plates were infected with OV-SY17 (multiplicity of infection [MOI], 1) and transfected with the pUC57-120LF-EGFP-120RF recombination plasmid. Recombinant viruses forming GFP-positive plaques were selected using fluorescence microscopy and further purified by limiting dilution and plaque purification. The OV-SY17Δ120 engineered mutant was confirmed by diagnostic PCR and DNA sequencing. In addition, the revertant plasmid pUC19-RV120 containing an N-terminal FLAG tag, the ORF120 coding sequence, and a red fluorescent protein (RFP) tag at the C terminus was used to transfect OFTu cells previously infected with OV-SY17Δ120. As described above, an RFP-tagged ORF120 revertant virus OV-SY17-RV120 was generated by homologous recombination and identified by sequencing. The recombinant viruses used in the study have been sequenced, and no difference other than the intended was found.

### ORFV ORF120 gene transcription kinetics.

The transcription kinetics of the ORFV ORF120 gene were determined by reverse transcription-PCR (RT-PCR). OFTu cells were mock infected or infected with OV-SY17 (MOI, 10) in the presence or absence of cytosine arabinoside (AraC; 40 μg/ml; Sigma-Aldrich, St. Louis, MO), an inhibitor of viral DNA replication and late gene expression, and harvested at 1, 2, 3, 6, 12, and 24 h postinfection (hpi). The mRNA level of ORFV ORF120 was detected by RT-PCR. In addition, the mRNA levels of ORFV127 (an early viral gene) and ORFV055 (a late viral gene) were measured in parallel controls. To determine whether the presence of foreign DNA (including GFP and its promoter) in the ORF120-deletion mutant has an impact on the transcription of ORF120 neighboring genes, the transcript levels of ORFV119 and ORFV121 were further detected by RT-PCR. In addition, the transcription of two neighboring genes of ORF120 in parental virus OV-SY17 and OV-SY17-Δ120 deletion mutant were also further detected by RT-PCR. The PCR primers used for the specific detection of ORFV ORF120, ORFV127, ORFV055, ORFV119, and ORFV121 were listed in Table 2.

### Characterization of ORFV ORF120 protein subcellular localization.

To evaluate the subcellular localization of the ORFV ORF120 protein, OFTu cells were mock infected or infected with OV-SY17-RV120 carrying the red fluorescent protein (RFP; MOI, 10) and harvested at the indicated times. The distribution of the viral ORF120 protein was observed by immunofluorescence. To further confirm the expression pattern of the ORF120 protein, OFTu and HeLa cells were transfected with pIRES-puro3-EGFP120 and pIRES-puro3-EGFP, respectively. At 24 h posttransfection, cell samples were collected and fixed for 15 min with 4% paraformaldehyde at room temperature (RT). After cells were washed three times with PBS, 4′,6-diamidino-2-phenylindole (DAPI) was used for nuclear fluorescence staining at RT for 10 min. Finally, the cells were washed and mounted for confocal microscopy.

### Analysis of the one-step and multistep viral growth kinetics.

To investigate the effect of ORF120 deletion on ORFV replication, OFTu cells were infected with parental virus OV-SY17, mutant virus OV-SY17Δ120, or revertant virus OV-SY17-RV120 (MOI, 0.1 or 10). Virus supernatants were harvested, and virus titers were determined by 50% tissue culture infectious dose (TCID_50_) assays at the indicated time points postinfection. One-step and multistep growth curves for the wild-type and recombinant viruses were generated, and the virus growth kinetics within infected cells were analyzed.

### Transcriptome and digital gene expression (DGE) analyses.

To determine whether the ORF120 deletion causes transcriptional changes in the host gene, OFTu cells were infected with the wild-type virus OV-SY17 (group 1) or the mutant virus OV-SY17Δ120 (group 2) with three duplicates (MOI, 10), and the differentially expressed genes in group 1 and group 2 were analyzed by transcriptome sequencing (RNA-seq). Briefly, OFTu cells infected with OV-SY17 and OV-SY17Δ120 were collected 3 h postinfection (hpi) and subjected to total RNA extraction with TRIzol reagent (Invitrogen, San Diego, CA). A total amount of 1 μg of RNA per sample was used as input material for the RNA sample preparations. cDNA synthesis, cDNA library construction, and Illumina sequencing on the Illumina HiSeq 2000 platform were performed by Biomarker Technologies Corporation (Beijing, China).

The raw reads in FASTQ format were first processed through in-house Perl scripts. The reads containing adapters and poly-Ns, as well as low-quality reads, were removed. All downstream analyses were based on clean data of high quality. Gene function was annotated based on the following databases: NCBI nonredundant protein sequences (Nr), NCBI nonredundant nucleotide sequences (Nt), protein family (Pfam), Clusters of Orthologous Groups of proteins (KOG/COG), Swiss-Prot (a manually annotated and reviewed protein sequence database), KEGG Ortholog database (KO), and Gene Ontology (GO).

### Real-time PCR validation for differentially expressed transcripts mediated by NF-κB and non-NF-κB pathways.

In response to ORF120 gene deletion, several proinflammatory cytokines and proinflammatory mediators mediated by NF-κB were screened out by digital gene expression profiling and transcriptome analysis. To confirm these results, OFTu cells were infected with OV-SY17 and OV-SY17Δ120 (MOI, 10) and harvested 1, 2, and 3 hpi. The mRNA levels of the proinflammatory cytokines (IL-1β and IL-8) and the proinflammatory mediators (NF-κBIA, ICAM1, and PTGS2) through the NF-κB pathway were evaluated by quantitative real-time PCR (qPCR) at 1, 2, and 3 hpi. In addition, the mRNA levels of some non-NF-κB-mediated genes, such as FGF21, SYTL3, TMEM100, and MET, were also measured by qPCR. The fold changes are shown relative to the levels of the OV-SY17 control group. The results are presented as the mean values of three independent experiments. A statistical analysis was performed using Student’s *t* test with GraphPad Prism 5.0 software (*, *P < *0.05; **, *P* < 0.01).

### NF-κB luciferase reporter assay.

The effect of the ORFV ORF120 protein on the transcription activity of NF-κB was investigated by a dual-luciferase reporter assay. OFTu cells and HeLa cells were seeded into 12-well plates and then cotransfected with NF-κB luciferase reporter pNF-κB-Luc (Clontech, Mountain View, CA), *Renilla* luciferase encoding plasmid pRL-TK (Promega, Madison, WI), and eukaryotic expression vector pIRES-puro3-EGFP120 or empty vector using Lipofectamine 3000 (Invitrogen, San Diego, CA) in triplicate. Twenty-four hours after transfection, the luciferase activity was measured using the dual-luciferase reporter assay system (Promega) according to the manufacturer’s instructions. Firefly luciferase activity was normalized to *Renilla* luciferase activity. As a positive control, after transfection for 24 h, OFTu cells were treated with lipopolysaccharide (LPS) (250 ng/ml; Invitrogen) for 6 h. In addition, to further demonstrate the effect of the ORFV ORF120 protein on NF-κB transcriptional activity in ORFV-infected cells, OFTu cells cotransfected with pNF-κB-Luc and pRL-TK were mock infected or infected with OV-SY17, OV-SY17Δ120, or OV-SY17-RV120 at an MOI of 10 and harvested 1 hpi. Luciferase activities were determined as described above and normalized to uninfected cells. The results are presented as the mean values of three independent experiments. A statistical analysis was determined by one-way analysis of variance (ANOVA) followed by Dunnett’s multiple-comparison test or unpaired two-sided *t* test for two-group comparisons.

### The regulatory effect of ORFV ORF120 protein on the NF-κB pathway.

The effect of OV-SY17 on the activation of the NF-κB signaling pathway was initially assessed by Western blotting. OFTu cells were mock infected or infected with OV-SY17; incubated for 0.5, 1, 2, 4, 8, 12, or 24 h; and then harvested. Total protein was extracted and determined using a bicinchoninic acid (BCA) protein assay kit (Pierce). In addition, cytoplasmic and nuclear protein fractions were isolated by nuclear and cytoplasmic protein extraction kits (Pierce, Rockford, IL, USA) according to the manufacturer’s instructions. Each sample (40 μg) was resolved by SDS-PAGE, transferred to a polyvinylidene difluoride (PVDF) membrane, and immunoblotted using primary antibodies against IκBα (number 10268-1-AP; Proteintech), phospho-IκBα (Ser32/36) (number 9246; Cell Signaling), NF-κB-p65 (number 3034; Cell Signaling), phospho-NF-κB-p65 (Ser536) (number 3033; Cell Signaling), phospho-IKKα/β (Ser176/180) (number 2697; Cell Signaling), IKKβ (number 8943; Cell Signaling), GAPDH (number 60004-1-lg; Proteintech), and histone H3 (number ab1220; Abcam). The blots were developed using a chemiluminescence assay (number MA0186; Meilunbio).

To further explore the regulatory effect of the ORF120 protein carried by ORFV on the NF-κB signaling pathway, OFTu cells were mock infected or infected with OV-SY17, OV-SY17Δ120, or OV-SY17-RV120 for 0.5, 1, and 2 h and harvested. The preparation of the total protein samples and the isolation of cytoplasmic and nuclear protein fractions were performed as described above. Western blot analyses were performed as previously described. Additionally, OFTu and HeLa cells were transiently transfected with plasmids encoding GFP-120 (pIRES-puro3-EGFP120) or GFP control (pIRES-puro3-EGFP). Twenty-four hours posttransfection, cell samples were harvested and the isolated cytoplasmic and nuclear protein fractions were subjected to Western blot analysis as previously described in order to detect the nuclear translocation of p65. The blots were incubated with primary antibodies against IκBα (no. 10268-1-AP; Proteintech), phospho-IκBα (Ser32/36) (number 9246; Cell Signaling), NF-κB-p65 (number 3034; Cell Signaling), phospho-NF-κB-p65 (Ser536) (number 3033; Cell Signaling), phospho-IKKα/β (Ser176/180) (number 2697; Cell Signaling), IKKβ (number 8943; Cell Signaling), GAPDH (number 60004-1-lg; Proteintech), histone H3 (number ab1220; Abcam), and GFP (number T0005; Affinity). Densitometric analysis of the immunoblots was performed with ImageJ software. Statistical analysis was performed using Student’s *t* test.

### The detection of NF-κB-p65 nuclear translocation by immunofluorescence assay.

An immunofluorescence assay was used to investigate the nuclear translocation of NF-κB-p65 in OFTu cells. OFTu cells were mock infected or infected with OV-SY17, OV-SY17Δ120, or OV-SY17-RV120 (MOI, 10) and harvested at the indicated times. Samples were fixed with 4% paraformaldehyde, washed three times with PBS, and permeabilized with 0.2% Triton X-100 for 10 min at RT. After blocking with 5% nonfat milk powder for 1 h at RT, the cells were incubated overnight with primary antibody against NF-κB-p65 (number 3034; Cell Signaling) at 4°C and then incubated with secondary antibody Alexa Fluor 488-labeled goat anti-rabbit antibody (number 4412; Cell Signaling) or Alexa Fluor 594-labeled goat anti-rabbit antibody (number 8889; Cell Signaling) for 1 h at 37°C. Finally, the cells were washed in PBS and stained with DAPI for 10 min at RT. The fluorescence images were inspected with a confocal laser scanning microscope. Cells from randomly selected fields totaling at least 400 cells per sample were counted for calculating the ratio of NF-κB nuclear translocation. The results were depicted as the mean percentage of NF-κB p65-positive cells at each time point. Data are triplicates from three independent experiments. Statistical analysis was performed using the Student’s *t* test.

### The DUAL membrane yeast two-hybrid system and coimmunoprecipitation assay revealed that the ORF120 protein interacted with G3BP1.

The DUAL membrane yeast two-hybrid system constructed by HiTech (Shanghai, China) was applied to screen ORFV ORF120 interaction proteins. The experimental procedures were carried out according to the manufacturer’s instructions. To investigate the interaction of the ORFV ORF120 protein and host factor G3BP1, OFTu cells were cotransfected with pIRES-puro3-EGFP120 and pCMV-N-myc-G3BP1 or either empty vector. The cells were harvested 24 h posttransfection and lysed. Protein extracts of the transfected cells were immunoprecipitated with mouse anti-myc antibody (number 60003-2-Ig; Proteintech) or mouse anti-GFP (number 66002-1-Ig; Proteintech). The antigen/antibody complex was incubated with protein A/G agarose beads (Thermo Fisher). Finally, the immunoprecipitates were washed three times with lysis buffer and then subjected to Western blotting. Furthermore, immunoprecipitation of G3BP1 was performed on OFTu cells infected with RFP-tagged ORF120 revertant virus using a rabbit anti-RFP antibody (number ab152123; Abcam), and the pcDNA3.1-RFP-Becline 1 plasmid was used as a control.

### The colocalization observation of the ORF120 protein and G3BP1 by confocal microscopy.

The interaction of the ORFV ORF120 protein and host factor G3BP1 was confirmed by confocal microscopy analysis. OFTu cells and HeLa cells were cotransfected with pIRES-puro3-EGFP120 and pcDNA3.1-mCherry-G3BP1 or an empty vector, respectively. The cells were harvested 24 h posttransfection and fixed with 4% paraformaldehyde for 15 min, washed three times with PBS, and stained with DAPI for 10 min at RT. Finally, the cells were washed and mounted for confocal microscopy.

Moreover, the colocalization of both the ORFV ORF120 protein and G3BP1 in the infected cells was evaluated. OFTu cells were mock infected or infected with OV-SY17Δ120 or OV-SY17-RV120 (MOI, 10). The cells were harvested at the indicated times and fixed with 4% paraformaldehyde, washed three times with PBS, and permeabilized with 0.2% Triton X-100 for 10 min at RT. After blocking with 5% nonfat milk powder for 1 h at RT, the cells were incubated overnight with a primary antibody against G3BP1 (number 13057-2-AP; Proteintech) at 4°C and then incubated with secondary antibody Alexa Fluor 488-labeled goat anti-rabbit antibody (number 4412; Cell Signaling) or Alexa Fluor 594-labeled goat anti-rabbit antibody (number 8889; Cell Signaling) for 1 h at 37°C. Finally, the cells were washed in PBS and stained with DAPI for 10 min at RT. Fluorescence images were inspected with a confocal laser scanning microscope.

### Identification of the binding domain of G3BP1.

To determine the binding domain of G3BP1 with the ORFV ORF120 protein, truncated mutants of G3BP1 (including myc-G3BP1^ΔNTF2^, myc-G3BP1^NTF2^, myc-G3BP1^ΔRRM^, myc-G3BP1^ΔRGG^, myc-G3BP1^PxxP+RRM^, myc-G3BP1^NTF2+RRM^, GFP-G3BP1^RRM+RGG^, and GFP-G3BP1^RGG^) were constructed according to the latest published literature (35). HEK293T cells were cotransfected with pIRES-puro3-EGFP-ORF120 or pCMV-N-HA-ORF120, different G3BP1 variants, or an empty vector. Twenty-four hours posttransfection, the cells were harvested and lysed. Protein extracts of the transfected cells were immunoprecipitated overnight with GFP or HA antibody at 4°C. The antigen/antibody complex was incubated with protein A/G agarose beads (Thermo Fisher). Finally, the immunoprecipitates were washed three times with lysis buffer and then loaded onto an SDS-PAGE gel for immunoblot analysis.

### Effect of ORFV ORF120 protein on host factor G3BP1 expression.

To explore the effect of the ORFV ORF120 protein on G3BP1 expression, OFTu cells were mock infected or infected with OV-SY17, OV-SY17Δ120, or OV-SY17-RV120 for 0.5, 1, and 2 h and harvested. Then, OFTu cells infected with OV-SY17 for 0.5, 1, 2, 4, 8, 12, and 24 h were harvested and examined. Furthermore, the OFTu and HeLa cells were transfected with pIRES-puro3-EGFP120 and pIRES-puro3-EGFP plasmids, respectively. Twenty-four hours posttransfection, the cell samples were harvested and subjected to Western blot analysis. The blots were incubated with primary antibodies against G3BP1 (number 13057-2-AP; Proteintech) and GAPDH (number 60004-1-lg; Proteintech). The blots were developed using a chemiluminescence assay (number MA0186; Meilunbio). Densitometric analysis of the immunoblots was performed with ImageJ software. Statistical analysis was determined by one-way ANOVA followed by Dunnett’s multiple-comparison test or unpaired two-sided *t* test for two-group comparisons.

### G3BP1 is involved in NF-κB activation induced by the ORFV ORF120 protein.

The siRNA sequences targeting G3BP1 were synthesized by Ribobio Technology (Guangzhou, China). The siRNA sequence targeting G3BP1 was 5′-TCAGAATCATAGCCCATAA-3′. OFTu cells were transfected with siRNA using Lipofectamine 3000 (Invitrogen, San Diego, CA) according to the manufacturer’s protocol. To determine whether G3BP1 is involved in NF-κB activation induced by ORF120, the effect of G3BP1 on ORF120-induced NF-κB pathway activation was investigated using G3BP1-specific siRNA. Briefly, OFTu cells transduced with G3BP1-specific siRNA were infected with OV-SY17 or OV-SY17Δ120 (MOI, 10) and harvested at 0.5 hpi and then the phosphorylation levels of IKKα/β, IκBα, and NF-κB-p65 in siRNA-G3BP1 cells infected with OV-SY17 and OV-SY17Δ120 were determined by Western blotting. Moreover, the correlation of G3BP1 expression with ORF120 was investigated by Western blotting using OFTu cells and HeLa cells transiently transfected with plasmids encoding GFP-120 (pIRES-puro3-EGFP120) or GFP control (pIRES-puro3-EGFP). Further densitometry data were normalized by the amount of GAPDH. The fold changes are shown relative to the levels of the GFP control group. The data are presented as the mean values of three independent experiments. Statistical analysis was performed using Student’s *t* test (*, *P < *0.05; **, *P* < 0.01). Finally, the effect of full-length G3BP1 and truncated G3BP1 on the transcription activity of NF-κB induced by ORF120 was investigated by a dual-luciferase reporter assay. Briefly, HEK293T cells were seeded into 12-well plates and then cotransfected with NF-κB luciferase reporter pNF-κB-Luc (Clontech, Mountain View, CA), *Renilla* luciferase-encoding plasmid pRL-TK (Promega, Madison, WI), eukaryotic expression vector pIRES-puro3-EGFP120, and mutants of G3BP1 or empty vector using Lipofectamine 3000 in triplicate. Twenty-four hours after transfection, the luciferase activity was measured using the dual-luciferase reporter assay system (Promega) according to the manufacturer’s instructions. Firefly luciferase activity was normalized to *Renilla* luciferase activity. Data are representative of three independent experiments. Statistical analysis was performed using Student’s *t* test.

### Animal inoculations.

Sixteen 4- to 5-month-old lambs were randomly divided into 4 groups (*n* = 4 per group), including 1 control group and 3 experimental groups. Under general anesthesia, the infected groups were inoculated with OV-SY17, OV-SY17Δ120, or OV-SY17-RV120 by scarification with virus inoculum containing 10^7.37^ TCID_50_/ml in the inferior lip. Lambs in control group were inoculated with phosphate-buffered saline (PBS) in the same manner. Then, the inoculated animals were monitored daily for at least 20 days. The characteristic clinical signs and Orf lesions of all of the groups were observed and recorded daily.

### Ethics statement.

All animal experiments in this study were approved by Institutional Animal Care and Use Committee of Jilin University and performed in accordance with the animal ethics guidelines and approved protocols.

### Statistics.

All data are presented as the means ± standard deviation (SD) from three sets of independent experiments. Graphs were created by Prism software version 5 (GraphPad Software, San Diego, CA, USA). Statistical analysis was performed with a one-way ANOVA followed by Dunnett’s multiple-comparison test or unpaired two-sided *t* test using GraphPad Prism software. The results were considered significant when the *P* value was <0.05.

### Data availability.

The data set containing the raw reads was deposited in the NCBI Sequence Read Archive (SRA) database (BioProject accession number PRJNA675661).
